# Signal transduction at GPCRs: Allosteric activation of the ERK MAPK by β-arrestin

**DOI:** 10.1073/pnas.2303794120

**Published:** 2023-10-16

**Authors:** Alem W. Kahsai, Kunal S. Shah, Paul J. Shim, Mason A. Lee, Bowie N. Shreiber, Allison M. Schwalb, Xingdong Zhang, Henry Y. Kwon, Li-Yin Huang, Erik J. Soderblom, Seungkirl Ahn, Robert J. Lefkowitz

**Affiliations:** ^a^Department of Medicine, Duke University Medical Center, Durham, NC 27710; ^b^Duke University School of Medicine, Duke University Medical Center, Durham, NC 27710; ^c^Department of Medicine, College of Medicine, The University of Arizona, Phoenix, AZ 85004; ^d^General Surgery Residency Program, Henry Ford Hospital, Detroit, MI 48202; ^e^Department of Cell Biology, Duke University Medical Center, Durham, NC 27710; ^f^Duke Center for Genomic and Computational Biology, Duke University Medical Center, Durham, NC 27710; ^g^Department of Biochemistry, Duke University Medical Center, Durham, NC 27710; ^h^Department of Chemistry, Duke University Medical Center, Durham, NC 27710; ^i^HHMI, Duke University Medical Center, Durham, NC 27710

**Keywords:** β-arrestin, scaffold proteins, signal transduction, catalytic scaffolds, ERK MAPK

## Abstract

β-arrestins are multivalent adaptor proteins that bind active phosphorylated G protein-coupled receptors (GPCRs) to inhibit G protein signaling, mediate receptor internalization, and initiate alternative signaling events. β-arrestins link agonist-stimulated GPCRs to downstream signaling partners, such as the c-Raf–MEK1–ERK1/2 cascade leading to ERK1/2 activation. β-arrestins have been thought to transduce signals solely via passive scaffolding by facilitating the assembly of multiprotein signaling complexes. Recently, however, β-arrestin 1 and 2 were shown to activate two downstream signaling effectors, c-Src and c-Raf, allosterically. Over the last two decades, ERK1/2 have been the most intensely studied signaling proteins scaffolded by β-arrestins. Here, we demonstrate that β-arrestins play an active role in allosterically modulating ERK kinase activity in vitro and within intact cells. Specifically, we show that β-arrestins and their GPCR-mediated active states allosterically enhance ERK2 autophosphorylation and phosphorylation of a downstream ERK2 substrate, and we elucidate the mechanism by which β-arrestins do so. Furthermore, we find that allosteric stimulation of dually phosphorylated ERK2 by active-state β-arrestin 2 is more robust than by active-state β-arrestin 1, highlighting differential capacities of β-arrestin isoforms to regulate effector signaling pathways downstream of GPCRs. In summary, our study provides strong evidence for a new paradigm in which β-arrestins function as active “catalytic” scaffolds to allosterically unlock the enzymatic activity of signaling components downstream of GPCR activation.

## Significance

β-arrestins function to “arrest” G protein signaling to promote GPCR desensitization and internalization. They also initiate a diverse set of signaling events, including ERK1/2 activation, once thought to only occur by passive scaffolding and formation of multiprotein “signalosomes.” Herein, we demonstrate that β-arrestins and their GPCR-bound active states allosterically enhance ERK2 kinase activity. Our findings reveal a previously unappreciated mechanism by which β-arrestins function both as passive scaffolds and allosteric regulators to modulate downstream GPCR signaling pathways.

β-arrestins (βarrs) are versatile adapter protein scaffolds that play central roles in G protein-coupled receptor (GPCR) desensitization, endocytosis, and signaling (1–4). The mammalian family of arrestin proteins consists of two ubiquitously expressed βarr isoforms, 1 and 2 (also known as arrestin-2 and -3, respectively), and two visual arrestins, arrestin-1 and -4, that bind rod and cone photoreceptors (1, 4, 5). βarrs were originally identified as proteins that sterically block G protein coupling to agonist-activated GPCRs, promoting receptor desensitization and internalization (3–7). The mechanism of desensitization involves phosphorylation of multiple sites, primarily within the carboxy-terminal tail of agonist-activated receptors by GPCR kinases (GRKs) (1, 3, 8, 9). Subsequently, βarrs are recruited to the phosphorylated receptor tail and the agonist-modified conformation of the receptor intracellular, transmembrane core, blocking G protein binding. Consequently, receptor activation of downstream G protein pathways is attenuated, and the receptor is targeted to clathrin-coated pits for endocytosis (10–12).

Canonical mitogen-activated protein kinase (MAPK) cascades are among the best-studied signaling pathways that regulate cellular responses to extracellular stimuli (13, 14). The MAPK cascade comprises three kinases that are activated sequentially: MAPKKK/MAP3K, MAPKK/MAP2K, and MAPK. Signal propagation through MAPK cascades is typically guided by distinct scaffolding proteins that assemble individual components together into spatially localized signaling complexes (13, 15–17). In mammalian cells, the extracellular signal-regulated kinase 1/2 (ERK1/2) is a well-characterized component of the MAPK pathway (13–15). The ERK1/2 pathway is activated via multiple mechanisms in response to extracellular stimuli, including growth factors, hormones, cytokines, and insulin, as well as intracellular processes and pathological conditions (13–15). Signaling through the canonical ERK1/2 pathway involves Ras-mediated activation of Raf (MAP3K), which activates MEK (MAP2K). Activated MEK catalyzes ERK1/2 (MAPK) phosphorylation within its activation loop at threonine and tyrosine residues of the conserved Thr–Glu–Tyr (TEY) motif (hereafter p-Thr^183^ and p-Tyr^185^ based on rat ERK2) (13, 14, 18, 19). Activated ERK1/2 executes downstream signal transduction by phosphorylating its nuclear and cytoplasmic substrates to control critical signaling nodes that regulate multiple cellular functions, including cell cycle progression, proliferation, survival, apoptosis, chemotaxis, neuronal differentiation, and synaptic changes underlying memory and learning (13, 14). Consequently, aberrant ERK1/2 activity occurs through multiple mechanisms and is observed in several human diseases, including cancer and neurodegenerative disorders (13, 14).

In addition to their function in attenuating GPCR-mediated G protein signaling, βarrs act as molecular scaffolds in assembling signaling complexes, including RAF–MEK–ERK mitogenic signaling pathway components (15, 16, 20–28). Recent studies have shown that βarrs act not simply as passive scaffolds but can directly enhance Src and Raf1 kinase activities through allosteric mechanisms (29–31). Similarly, in the yeast mating pheromone response MAPK pathway (Ste11–Ste7–Fus3), the scaffold protein Ste5 was shown to allosterically regulate the activity of the MAPK Fus3, a homolog of mammalian ERK1/2 (17, 32, 33). Although scaffolds such as βarrs are generally known to enhance signal transduction efficiency, little is understood about the mechanisms by which they regulate ERK1/2 activity in mammals. Herein, we show that βarrs and their GPCR-mediated active states allosterically activate ERK2. Thus, βarrs function not only as passive scaffold platforms for assembly of the Raf–MEK–ERK cascade but also as active positive allosteric modulators of ERK when coupled to agonist-activated phosphorylated GPCRs.

## Results

### The Scaffold Proteins β-arrestin 1/2 Interact with ERK2.

**Fig. 1.**
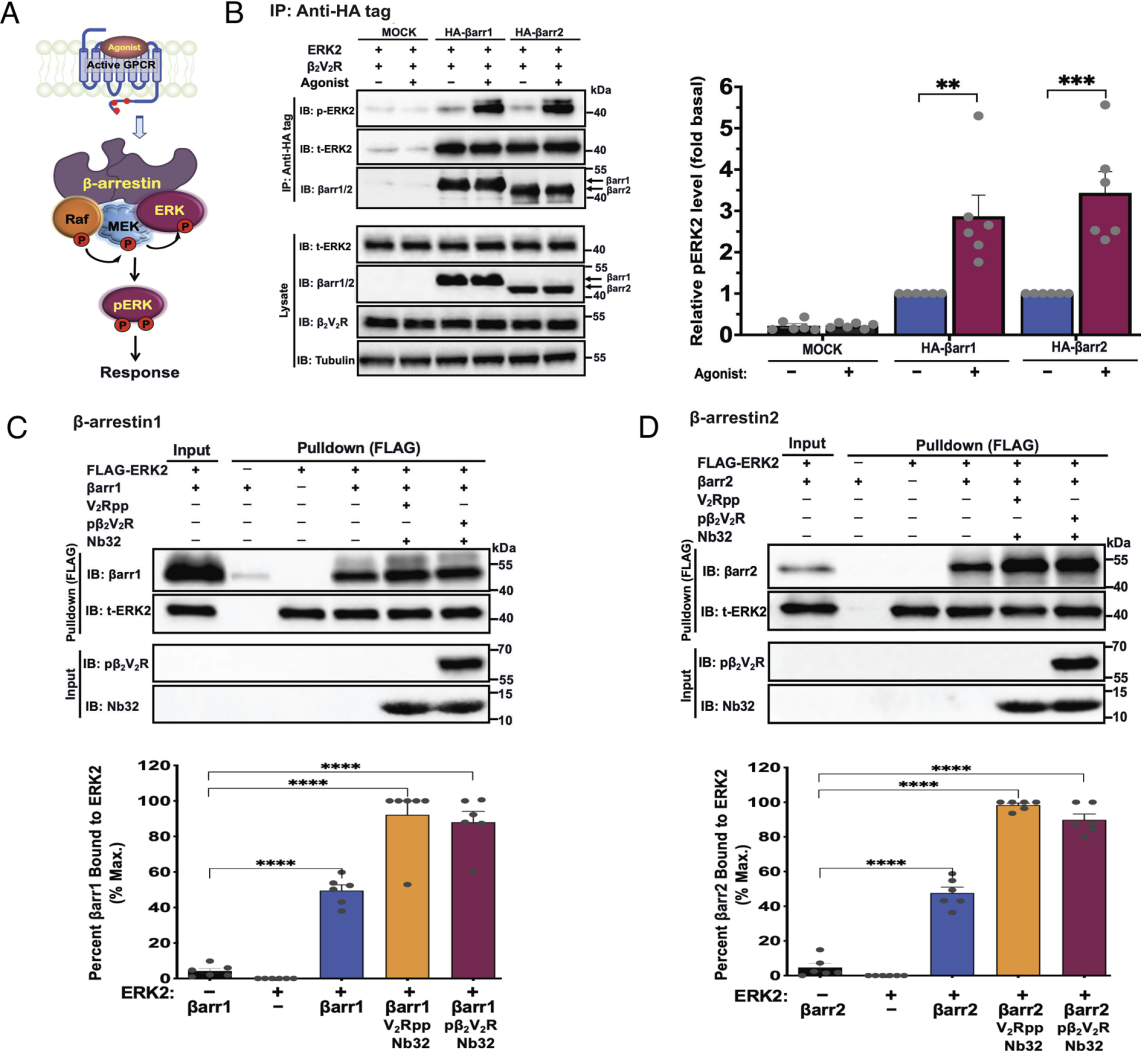
The scaffold proteins β-arrestin1 and 2 interact with ERK2. (*A*) Cartoon showing a model of a βarr scaffold linking agonist-activated phosphorylated receptor with components of the RAF–MEK–ERK MAPK pathway, which ultimately leads to ERK activation. (*B*) βarr1 and 2 form complexes with ERK2 as assessed by coimmunoprecipitation. CRISPR/Cas9 βarr1/2-knockout HEK293 cells were transfected with empty vector or HA-tagged βarr1 or 2 plus β_2_V_2_R and ERK2 expression plasmids. After 48 h, cells were serum-starved and subsequently stimulated with the β_2_AR agonist isoproterenol (10 μM), lysed, and immunoprecipitated with anti-HA antibody affinity gel. Precipitated protein complexes were detected by western blotting using anti-phospho-ERK1/2 (anti-ERK2 pThr^183^/pTyr^185^ for rat species), total ERK2, or anti‐HA-HRP (βarr1/2) antibodies. Total ERK2, βarr1/2, β_2_V_2_R, and tubulin immunoblots are shown as input controls. Bar graphs (*Right*) illustrate quantifications of phospho-ERK2 levels. (*C* and *D*) ERK2 binds βarr1/2 and their receptor-mediated active states. Pulldown of βarr1 (*C,* *Top*) or βarr2 (*D,* *Top*) with FLAG-tagged ERK2 on anti-FLAG beads. Purified 3xFLAG-tagged ERK2 was incubated with βarr1 or 2 alone or each in their active state form bound to V2Rpp or pβ2V2R:BI-167107 together with active βarr-stabilizing nanobody (Nb32). The pull-down products were analyzed by western blotting with antibodies specific for total ERK2 or βarr1/2 as shown by representative blots. βarr1/2, ERK2, pβ_2_V_2_R, and Nb32 input control immunoblots are shown. Graphs (*Bottom*) show quantification levels of βarr1/2. Data are means ± SEM of at least five independent experiments. **P ≤ 0.01; ***P ≤ 0.001; and ****P ≤ 0.0001 (ANOVA).

βarrs have been shown to function as scaffolds by assembling components of the prototypical MAPK ERK cascade (c-Raf–MEK1/2–ERK1/2), leading to ERK1/2 activation (Fig. 1*A*) (15, 16, 20–23). Here, we set out to investigate whether βarrs in their free basal or active states can regulate ERK2 kinase activity through an allosteric mechanism. To test this possibility, we examined the interaction between βarr1 or 2 and ERK2 in an agonist-mediated receptor activation-dependent manner. Accordingly, we performed coimmunoprecipitation experiments in CRISPR/Cas9 βarr1/2 knockout HEK293 cells transfected with plasmids expressing hemagglutinin (HA)-tagged βarr1 or 2 plus ERK2 and the β_2_V_2_R chimeric receptor that binds βarrs with higher affinity compared to wild-type β_2_AR (34–38). As presented in Fig. 1*B*, βarr1/2 was coimmunoprecipitated with ERK2 and phosphorylated ERK2. This mechanism was further enhanced following agonist-mediated stimulation of the receptor. These results were corroborated in vitro using purified proteins to monitor direct interaction between ERK2 and βarrs in their free or active state forms (bound to phosphorylated receptor [pβ_2_V_2_R] or its tail surrogate phosphopeptide [V_2_Rpp] with βarr-active state stabilizing nanobody, Nb32) (*SI Appendix*, Fig. S1) (35–39). As illustrated in Fig. 1 *C* and *D*, βarr1 and 2 were pulled down with FLAG-tagged ERK2, indicating that both βarr isoforms directly interact with ERK2. βarrs activated by V_2_Rpp or pβ_2_V_2_R have further enhanced association with ERK2, compared to free βarr. These results demonstrate that although βarr binding to ERK increases moderately in the presence of a phosphopeptide or phosphorylated receptor, βarrs can interact with ERK2 even in the absence of a phosphorylated receptor.

### β-arrestins and Their Active States Allosterically Stimulate ERK Autophosphorylation.

**Fig. 2.**
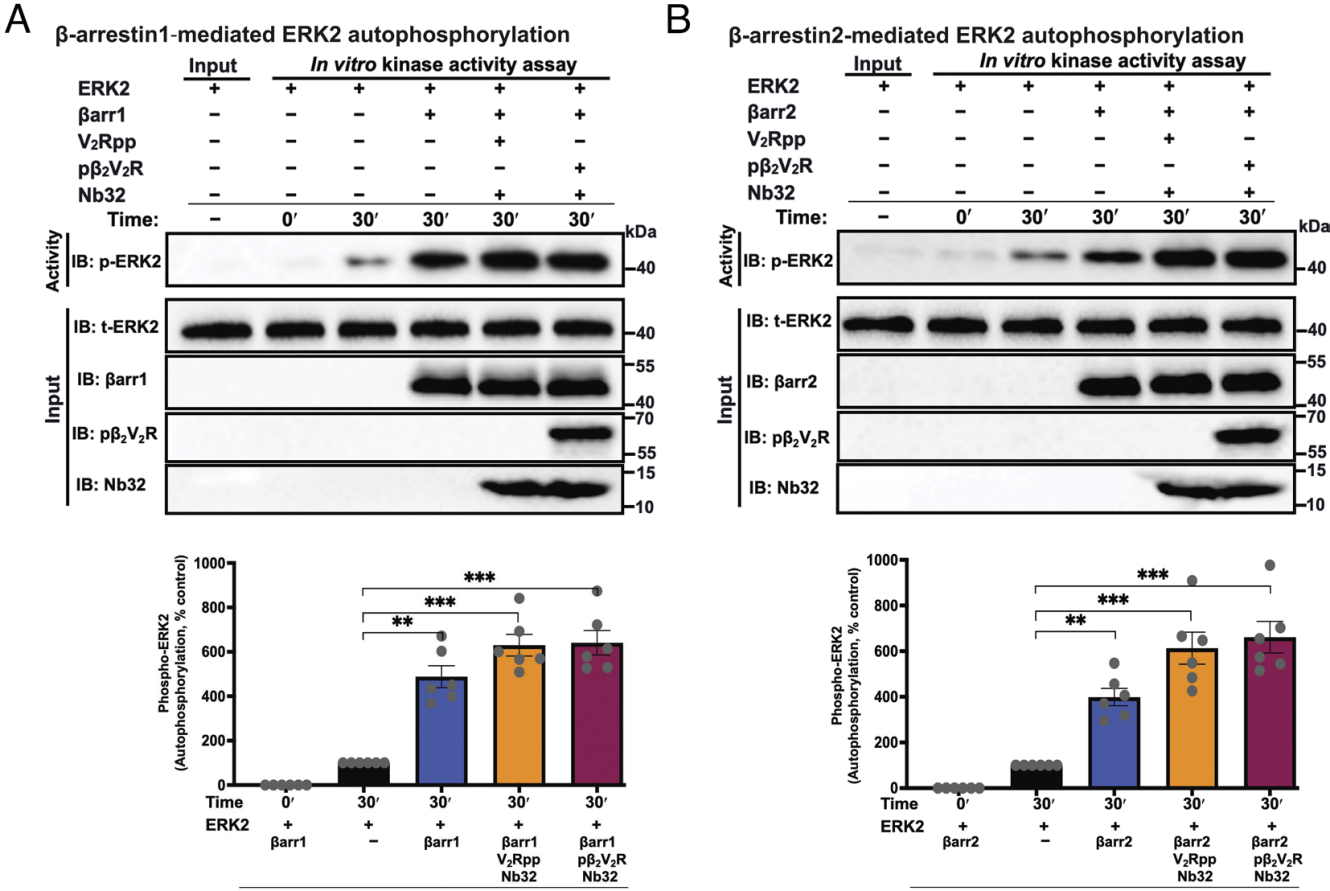
β-arrestin1/2 and their active states allosterically stimulate ERK2 autophosphorylation. (*A* and *B*, *Top*) shows representative western blots demonstrating that βarr1/2 and their active states (bound to V_2_Rpp or pβ_2_V_2_R) stimulate the autophosphorylation activity of ERK2. Reactions were performed in an endpoint format (30 min) at 30 °C using ERK2 (30 nM) with or without 1 μM βarr1 (*A*) or βarr2 (*B*) or each bound to V_2_Rpp or pβ_2_V_2_R together with Nb32. Samples were subsequently analyzed by western blotting with antibody specific for phospho-ERK1/2 (anti-ERK2-pT^183^/pY^185^). Total ERK2, βarr1/2, pβ_2_V_2_R, and Nb32 immunoblots represent individual input controls. Lower panels in *A* and *B* show bar graphs representing the extent of ERK2 autophosphorylation under the different conditions expressed as the average fold enhancement (means ± SEM) relative to a control reaction done with buffer (i.e., ERK2 alone treated as 100%). Data shown are means ± SEM (N = 6); significances by one-way ANOVA, followed by Dunnett's multiple comparison test. **P ≤ 0.01, ***P ≤ 0.001, and ****P ≤ 0.0001.

To examine the allosteric effect of βarr on ERK2 activity, we investigated how βarrs influence ERK autophosphorylation in an in vitro experimental setting that excluded upstream cascade components (c-Raf and MEK1). Therefore, we performed in vitro kinase assays to determine autophosphorylation of ERK2 alone, as assessed by immunoblotting using a phospho-ERK1/2 antibody, with increasing ERK2 concentrations (*SI Appendix*, Fig. S2). We found that ERK2 could phosphorylate itself linearly with regard to ERK concentration, suggesting a single phosphorylation event. Having demonstrated that ERK can phosphorylate itself, we next examined whether basal state βarr1/2 or their active state forms (bound to either V_2_Rpp or pβ_2_V_2_R) can allosterically regulate ERK2 autophosphorylation activity in the endpoint format kinase assay assessed by immunoblotting (Fig. 2). As shown in Fig. 2 *A* and *B*, binding of βarrs to ERK2 robustly enhances its autophosphorylation activity, both in their basal states and more robustly in their active states.

**Fig. 3.**
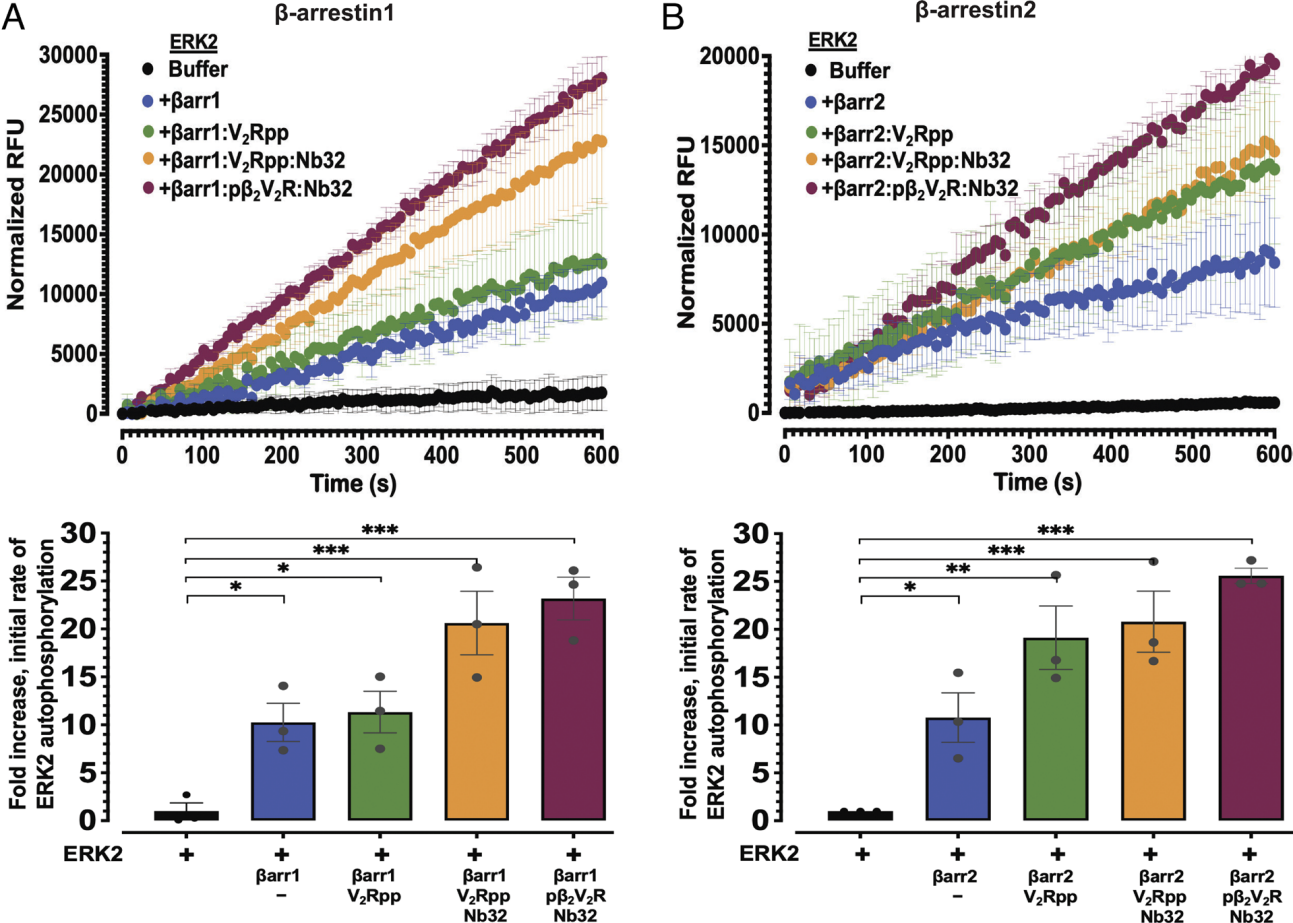
β-arrestin1/2 allosterically enhance the rate of ERK2 autophosphorylation. (*A* and *B*) show ERK2 autophosphorylation kinetics assessed using an in vitro real-time fluorescence-based kinase assay, in the absence or presence of basal state βarr1 (*A*) or βarr2 (*B*) or each in their active state forms bound to either V_2_Rpp or pβ_2_V_2_R together with Nb32. Kinase reactions were carried out with ERK2 (15 nM) and βarr1/2 (300 nM). Graphs (*Top*) represent the time course transitions of ERK2 autophosphorylation in relative fluorescence units (RFUs). Each experiment included control wells lacking ERK2 (with βarr 1 or 2, and ATP), and fluorescence signal data points from control reactions were subtracted to obtain corrected RFUs. (*A* and *B*, *Lower*) show quantification of ERK autophosphorylation presented as fold-enhancement of initial rates from each profile relative to vehicle control (ERK2 alone treated as onefold). Mean values are plotted, with error bars representing SEM (N = 3). Significances by one-way ANOVA, followed by Dunnett's multiple comparison test. *P ≤ 0.05; **P ≤ 0.01; and ***P ≤ 0.001.

Given the robust enhancement of ERK2 autophosphorylation by βarrs as assessed by immunoblotting, we sought to obtain quantitative information about ERK kinase activity by a complementary approach. We utilized a fluorescent-based real-time in vitro kinase activity to investigate the kinetics of βarr-stimulated ERK2 autophosphorylation (*SI Appendix*, Fig. S3*A*). The fluorescent-based real-time assay monitors Adenosine triphosphate (ATP) consumption by a kinase via measuring Adenosine diphosphate (ADP) formation using an ADP-specific fluorescence sensor. To quantify the allosteric effect of βarrs on ERK2 activity, we calculated initial rates from time-course ERK2 activity profiles (*SI Appendix*, Fig. S3*B*). As shown in *SI Appendix*, Fig. S3*C*, the initial rates of ERK2 autophosphorylation activity are concentration-dependent, further demonstrating the utility of this assay for measuring real-time ERK2 activity in vitro. We next tested the effects of βarr1 or 2 alone or in their active state forms bound to either V_2_Rpp or pβ_2_V_2_R on ERK2 activity in this real-time in vitro kinase assay (Fig. 3). Analysis of the kinetic profiles revealed that both βarr1 and 2 significantly enhanced the ERK2 autophosphorylation rate (10.1 ± 2.6-fold and 10.7 ± 3.3-fold, respectively over control) (Fig. 3 *A* and *B*). Active states of βarr1 resulted in a much greater enhancement of ERK2 autophosphorylation compared to control (20.6 ± 3.3-fold and 23.2 ± 2.2-fold increases for βarr1–V_2_Rpp–Nb32 and βarr1–pβ_2_V_2_R complexes, respectively, over control, ERK2 alone) (Fig. 3*A*). Similarly, the active states of βarr2 stimulated a robust allosteric effect on ERK2 autophosphorylation (20.8 ± 3.1-fold and 25.6 ± 0.8-fold increases for βarr2–V_2_Rpp–Nb32 and βarr2–pβ_2_V_2_R complexes, respectively, over control, ERK2 alone) (Fig. 3*B*). Notably, unlike the V_2_Rpp-bound active state βarr2, activation of βarr1 by V_2_Rpp did not further enhance ERK2 activity. Greater enhancement required stabilization of βarr1–V_2_Rpp complex by Nb32. In contrast, there was no measurable difference between βarr2 bound to V_2_Rpp compared to the active state stabilized by Nb32, highlighting the ability of phosphorylated receptors to promote maximal activation of βarr2 and robust ERK2 activation. Collectively, these data show that βarrs allosterically regulate ERK2 activity.

### β-arrestins Allosterically Regulate ERK2 Activity by Promoting Tyrosine Autophosphorylation in its Activation Loop.

**Fig. 4.**
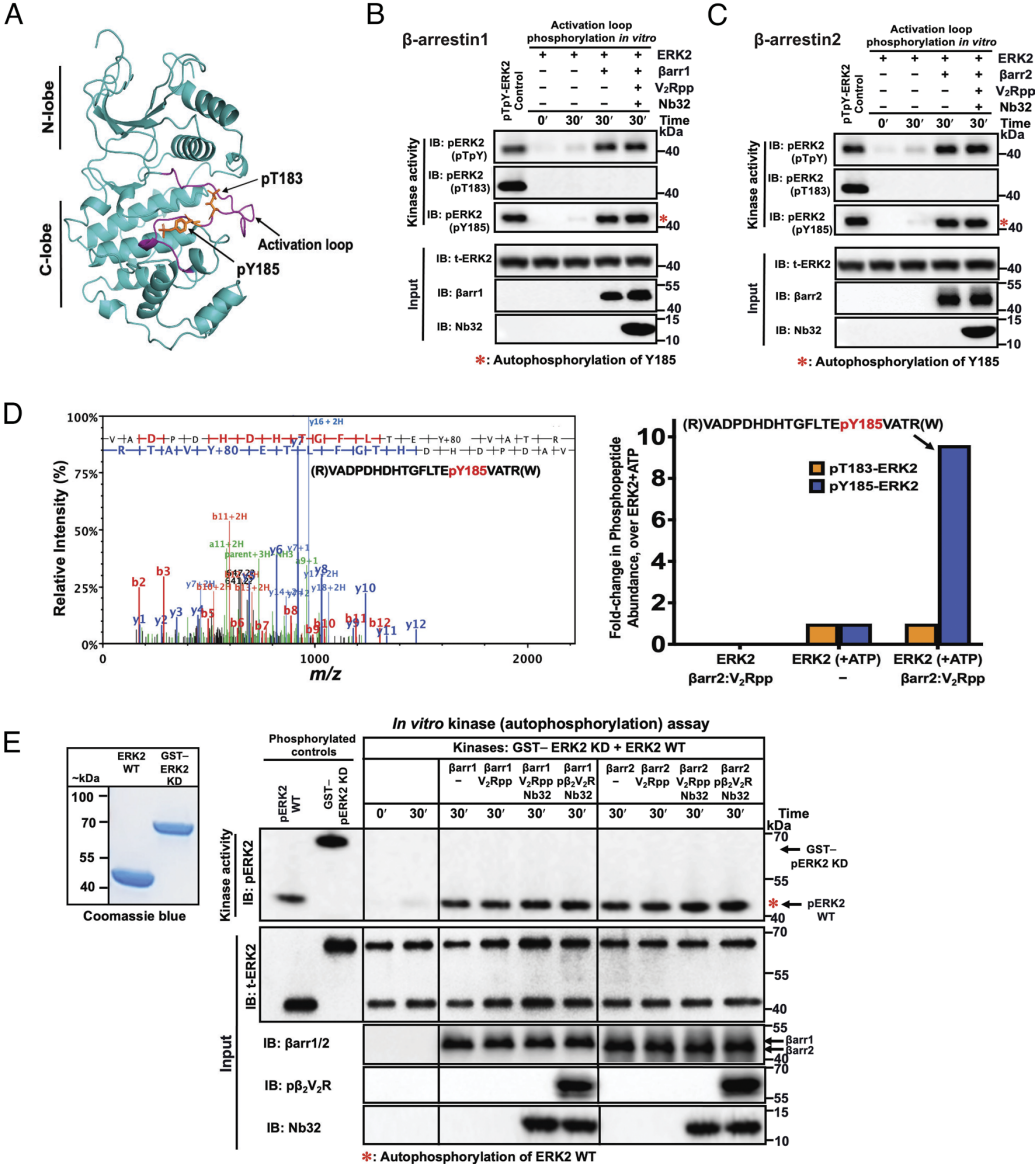
Binding of β-arrestins to ERK2 enhances autophosphorylation of a tyrosine residue in its activation loop and occurs intramolecularly (in *cis*). (*A*) Ribbon diagram of ERK2 (PDB code: 2ERK as template; illustration using PyMOL v.2.5 Schrödinger, LLC) showing kinase activity regulatory sites (pT^183^ and pY^185^ for rat ERK2 sequence) within the activation loop (in purple) of active ERK2 (18, 40). Phosphorylation on Thr^183^ locks catalytic residues in a productive conformation, while on Tyr^185^ enables substrate binding and specificity by influencing the conformation of the MAPK. (*B*) and (*C*) show representative immunoblots from an in vitro kinase assay examining activation loop autophosphorylation of ERK2 (30 nM) performed in the absence or presence of βarr1/2 (1 μM) or their active states bound to V_2_Rpp together with Nb32 at 30 °C for 30 min. Immunoblot analysis was performed using three activation loop site-specific phospho-ERK1/2 antibodies (against dual sites: anti-ERK2-pThr^183^/pTyr^185^; anti-ERK2-pThr^183^; or anti-ERK2-pTyr^185^). Total ERK2, βarr1/2, and Nb32 immunoblots are shown as input controls. (*D*) LC-MS/MS analysis validates that βarr-modulation produces a monophosphorylated ERK2 at Tyr^185^. MS analysis of the autophosphorylation reaction containing ERK2 with or without βarr2 bound to V_2_Rpp was tryptic digested and analyzed by LC-MS/MS. (*Left*) Tandem MS spectrum of the identified precursor phosphopeptide ion shows N- (“b” ions) and C- (“y” ions) terminus fragments indicating the phosphorylation of rat ERK2 Tyr residue at position 185 (presented above the spectrum). (*Right*) bar graphs show fold change in the relative abundance of pThr^183^ and pTyr^185^ containing phosphopeptides derived from the kinase reaction containing ERK2 alone (control treated as 1) or ERK2 treated with active βarr2–V_2_Rpp (*E*) βarr-stimulated Tyr^185^ ERK2 activation loop autophosphorylation occurs in *cis*. (*Left*) shows Coomassie stained gel of wt-ERK2 and GST-tagged kinase-dead (KD) ERK2. (*Right*) shows representative western blots from in vitro kinase assay performed by incubating KD-ERK2 (GST–ERK2-K52A; 45 nM) and wt-ERK2 (30 nM) together in the presence or absence of βarr1/2 (1 μM) or indicated active states and allowed to react for 30 min at 30 °C. Immunoblotting was performed using phospho-specific antibodies of ERK2. Inputs used for kinase reaction were analyzed by immunoblotting for total ERK2, βarr1/2, pβ_2_V_2_R, and Nb32. Dually phosphorylated wt-ERK2 (lane 1, ppERK2) and KD-ERK2 (lane 2, GST–ppERK2-K52A) were prepared by coincubating with active MEK1 are shown as positive controls. Representative blots of three independent experiments are shown.

To delineate the underlying mechanism of ERK2 stimulation by βarr, we sought to identify the phosphorylation site(s) in ERK2 subject to such modulation. As with most protein kinases, ERK2 activity is regulated by the phosphorylation state of residues within the activation loop “TEY” motif (13, 18, 19, 40) (Thr^183^ and Tyr^185^ residues of rat ERK2; Fig. 4*A*). Hence, we performed an in vitro ERK autophosphorylation assay in the absence or presence of βarrs in their basal or active states. Immunoblotting analysis using phosphorylation site-specific antibodies that recognize only either pThr^183^ or pTyr^185^ within the ERK2 activation loop showed that ERK2 autophosphorylation occurs at pTyr^185^ and that βarrs robustly enhanced this activity (Fig. 4 *B* and *C*). In contrast, anti-pThr^183^ antibody revealed no detectable phosphorylation of Thr^183^. To further validate the site of ERK2 autophosphorylation regulated by βarrs, we carried out in vitro kinase reactions with ERK2 alone or ERK2 incubated with active βarr2 bound to V_2_Rpp and analyzed tryptic digested peptides by liquid chromatography-tandem mass spectrometry (LC-MS/MS) (Fig. 4*D*). Consistent with our immunoblot analysis, LC-MS/MS analysis revealed that βarr enhanced ERK2 monophosphorylation at Tyr^185^. Additionally, the Tyr^185^ phosphorylation level in the ERK2 sample treated with active βarr2 was roughly ninefold higher than that in the absence of βarr2. This robust allosteric stimulation of ERK2 autophosphorylation activity by βarr agrees with our findings using a complementary immunoblotting approach (Figs. 2 and 4 *B* and *C*).

### Allosteric Regulation of ERK2 Autoactivation by βarrestin Occurs Intramolecularly (in *cis*).

To gain further insight into the mechanistic determinants of the allosteric effect of βarrs on ERK2 activity, we next investigated whether Tyr-autophosphorylation occurs intramolecularly (*i.e*., in *cis*) or intermolecularly (*i.e.,* in *trans*) (Fig. 4*E*). Kinase reactions were performed by coincubating wild-type (wt) ERK2 and N-terminal glutathione S-transferase (GST)-tagged kinase-dead (KD) ERK2 mutant (GST–ERK2-K52A), which allowed us to distinguish them by SDS-PAGE (Fig. 4 *E*, *Left*). Experiments were performed as above with or without βarr1 or 2 or their active states to determine their effects on Tyr-autophosphorylation. We expected that if Tyr-phosphorylation occurred intramolecularly, only wt-ERK2 would be phosphorylated; if phosphorylation occurred intermolecularly, both wt-ERK2 and KD-ERK2 would be phosphorylated. As shown in Fig. 4 *E* (*Right*), Tyr^185^ phosphorylation occurred on wt-ERK2 but not on KD-ERK2, indicating that Tyr-autophosphorylation occurs in *cis* via an intramolecular mechanism.

### Monophosphorylation of ERK2 on Y185 Substantially Increases Kinase Activity.

**Fig. 5.**
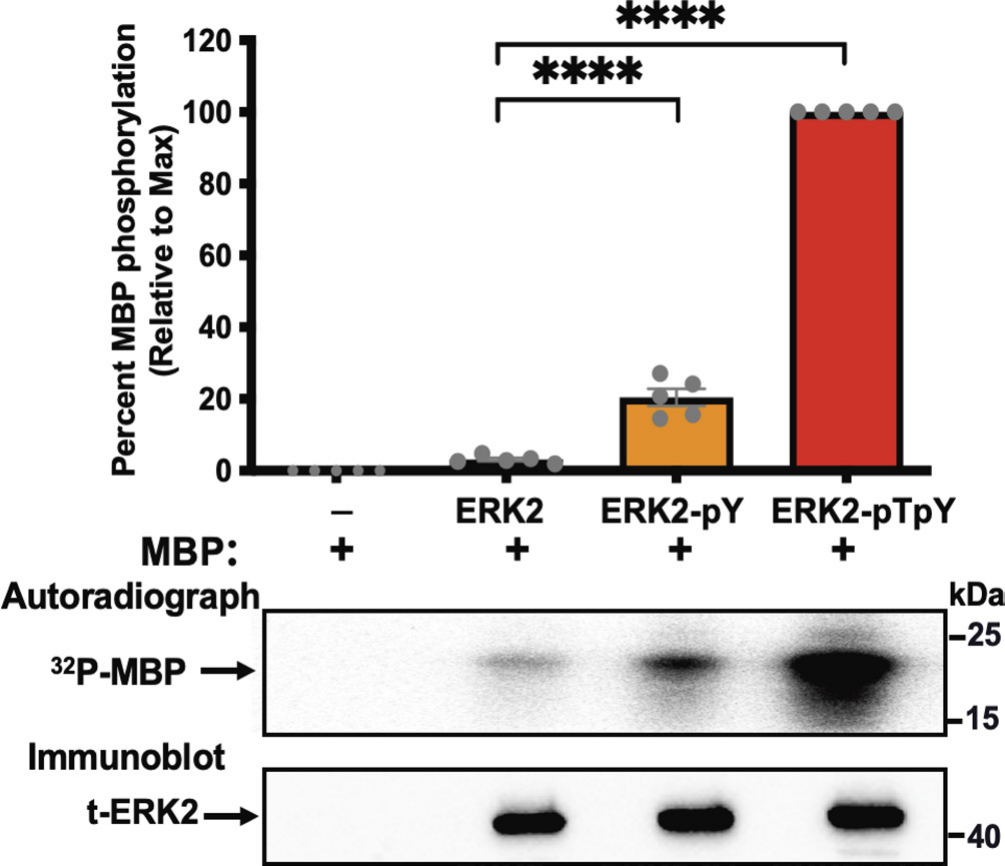
Monophosphorylation of ERK2 on Tyr185 substantially increases kinase activity. Bovine myelin basic protein (MBP) was incubated with ERK2 that was either unphosphorylated (ERK2), mono-phosphorylated ERK2 on Tyr^185^ (ERK2-pY), or dually phosphorylated on Thr^183^ and Tyr^185^ (ERK2-pTpY). Recombinant proteins were incubated at 37 °C for 1 h with [γ-^32^P] ATP as a phosphate source. Samples were separated on SDS-PAGE and ^32^P incorporation was assessed by autoradiography using a PhosphorImager. Total ERK2 immunoblot is shown as input control for each ERK2 species. Relative quantification of ^32^P incorporation is shown in the upper panel. Data are means ± SEM (N = 5). Statistical test: one-way ANOVA with Tukey's multiple comparison test, ****P ≤ 0.0001.

Next, we examined the functional consequences of βarr-stimulated monophosphorylation of ERK2 in the activation loop Tyr^185^ residue (ERK2-pY). This was accomplished by examining ERK2-pY activity towards myelin basic protein (MBP), one of its common substrates, in a kinase reaction using [γ-^32^P]ATP as a radioactive phosphate source (Fig. 5). As controls, we used the kinase activity of unphosphorylated ERK2 and dually phosphorylated ERK2 on Tyr^185^ and Thr^183^ (ERK2-pTpY). Monophosphorylated ERK2-pY was produced via an in vitro ERK2 autophosphorylation reaction in the presence of GST–βarr1 (*SI Appendix*, Fig. S4). Dually phosphorylated ERK2-pTpY was prepared in a kinase reaction by coincubating ERK2 with active human MEK1 (*SI Appendix*, Fig. S4). Subsequently, these ERK2 variants were purified using size-exclusion chromatography (SEC), and their phosphorylation status was assessed by immunoblotting using site-specific phospho-antibodies (*SI Appendix*, Fig. S4). Analysis of the kinase activities of these ERK2 variants shows that unphosphorylated ERK2 exhibited minimal activity toward MBP (Fig. 5). On the other hand, monophosphorylated ERK2-pY exhibited increased kinase activity toward MBP, albeit to a lesser extent than the dually phosphorylated ERK2-pTpY (~20% of ERK2-pTpY and ~7.2-fold over that of unphosphorylated ERK2). These findings suggest that the βarr-mediated Tyr^185^ phosphorylated form of ERK2 exhibits substantial kinase activity compared to unphosphorylated ERK2 and may likely serve as an initial priming of ERK2 for subsequent dual phosphorylation to generate the fully phosphorylated active form of ERK2.

### β-arrestins Allosterically Enhance the Activity of ERK2-pTpY to Phosphorylate MBP.

**Fig. 6.**
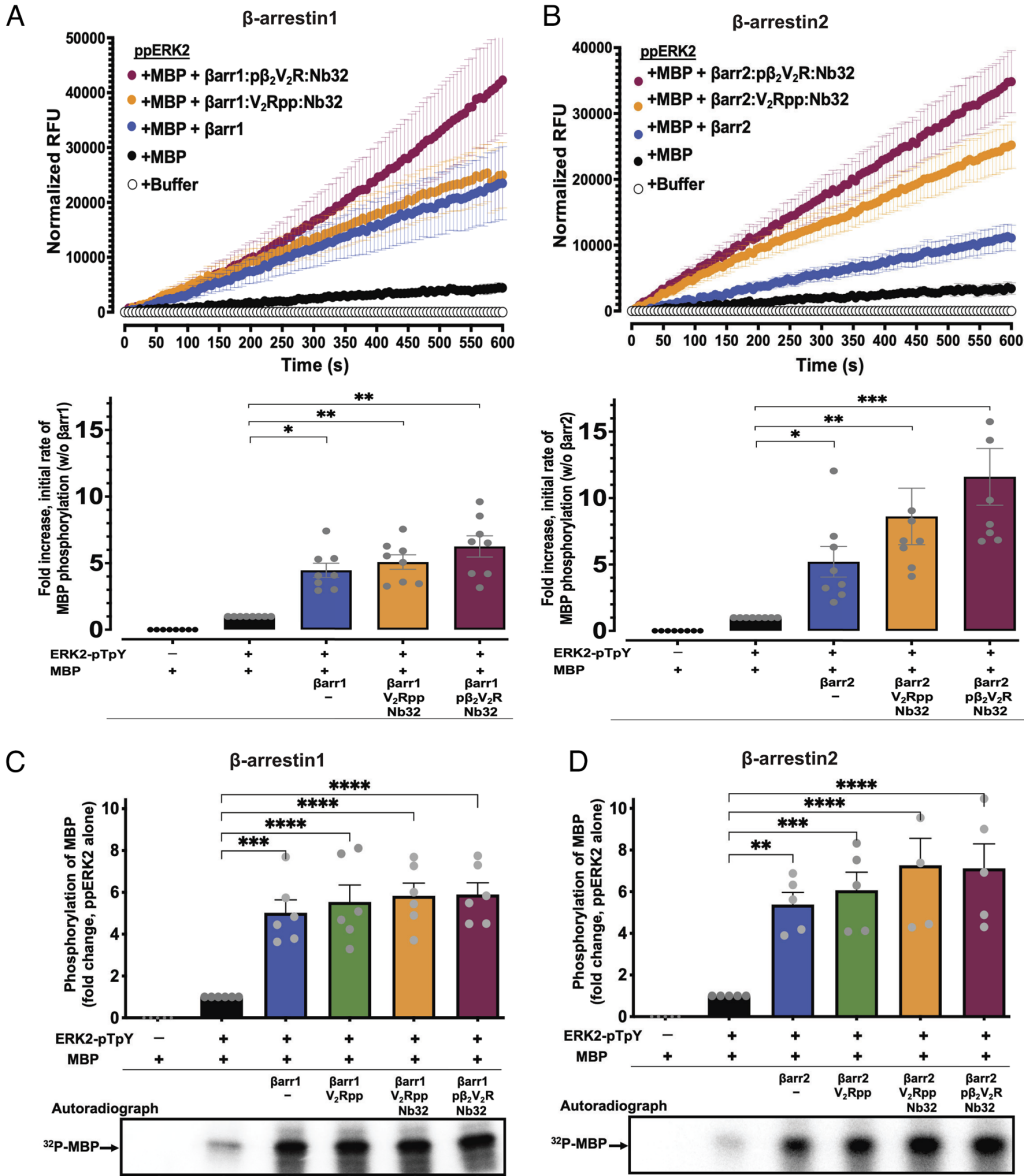
β-arrestins enhance the activity of dually phosphorylated ERK2-pTpY to phosphorylate MBP. (*A* and *B, Top* panels) Time courses of ERK2-pTpY-catalyzed phosphorylation of MBP as assessed using real-time fluorescence-based kinase assay in the absence or presence of basal state βarr1 (*A*) or βarr2 (*B*) or each in their active state forms (bound to V_2_Rpp or pβ_2_V_2_R together with Nb32). Relative fluorescence unit (RFU) intensities were background corrected to account for any contribution from control reactions: ERK2-pTpY alone and ERK2-pTpY with βarr1/2. Corrected RFU values are plotted as a function of time. Initial rates were determined from curve fitting of the linear phase of the reactions using linear regression. (*A* and *B*, *Lower* panels) quantification of phosphorylation of MBP presented as fold-enhancement of initial rates from each profile (as shown in the *Top* panel) relative to vehicle control (absence of βarr treated as onefold). Error bars indicate ± SEM of the mean from at least seven independent experiments. (*C* and *D*) Endpoint format kinase activity of ERK2-pTpY- against MBP was performed using [γ-^32^P] ATP as phosphate source in the absence or presence of βarr1 (*C*) or βarr2 (*D*) or their active state forms. Samples were separated on SDS-PAGE and ^32^P incorporation was assessed by autoradiography using a PhosphorImager. (*C* and *D, Top* panels) represent bar graphs of quantifications of phosphorylation levels of MBP under the indicated conditions. Lower panels in *C* and *D* show representative autoradiograms. Data are means ± SEM of at least five independent experiments. Asterisks indicated the significant difference based on one-way ANOVA analysis with Dunnett’s multiple comparison test (*P ≤ 0.05, **P ≤ 0.01, ***P ≤ 0.001, and ****P ≤ 0.0001).

Building on the observation that βarrs could enhance ERK2 autophosphorylation, we next investigated whether βarrs and their active state forms can modulate the kinase activity of dually phosphorylated ERK2-pTpY towards MBP (Fig. 6). To this end, we first measured the activity of dually phosphorylated ERK2-pTpY towards MBP with or without βarrs or their active states *via* the fluorescence-based quantitative real-time in vitro kinase assay (Fig. 6 *A* and *B*). As shown in Fig. 6 *A* and *B*, both βarr1 and 2 significantly enhanced ERK2-pTpY activity towards MBP (~fivefold increase relative to no βarr control). βarr1 activation upon binding to V_2_Rpp or phosphorylated receptor led to only a modest enhancement of activity over control (~sixfold compared to no βarr1 control) (Fig. 6*A*). On the other hand, βarr2 activation by binding to V_2_Rpp or phosphorylated receptor resulted in much greater enhancement of ERK2-pTpY activity towards MBP (~9.5-fold and 12-fold, respectively, compared to no βarr2 control) (Fig. 6 *A* and *B*). Of note, it was previously reported that βarr itself can be an ERK substrate (41). To prevent confounding from such phosphorylation in our fluorescence-based in vitro assay, we included appropriate controls in which MBP substrate was omitted. Such controls revealed only trivial phosphorylation of βarr under our experimental conditions (*Materials and Methods*). Interestingly, results from our fluorescence-based kinase assay indicate that active state βarr2 exerts a more substantial allosteric effect on ERK2-pTpY activity compared with the equivalent active state form of βarr1 isoform. Further confirming these results, we found that βarrs robustly stimulate the kinase activity of dually phosphorylated ERK2-pTpY in terms of its ability to phosphorylate MBP as probed by a complementary approach in which ^32^P incorporation into MBP was visualized by autoradiography (Fig. 6 *C* and *D*).

### β-arrestin–Mediated ERK2 Activation in Intact Cells.

**Fig. 7.**
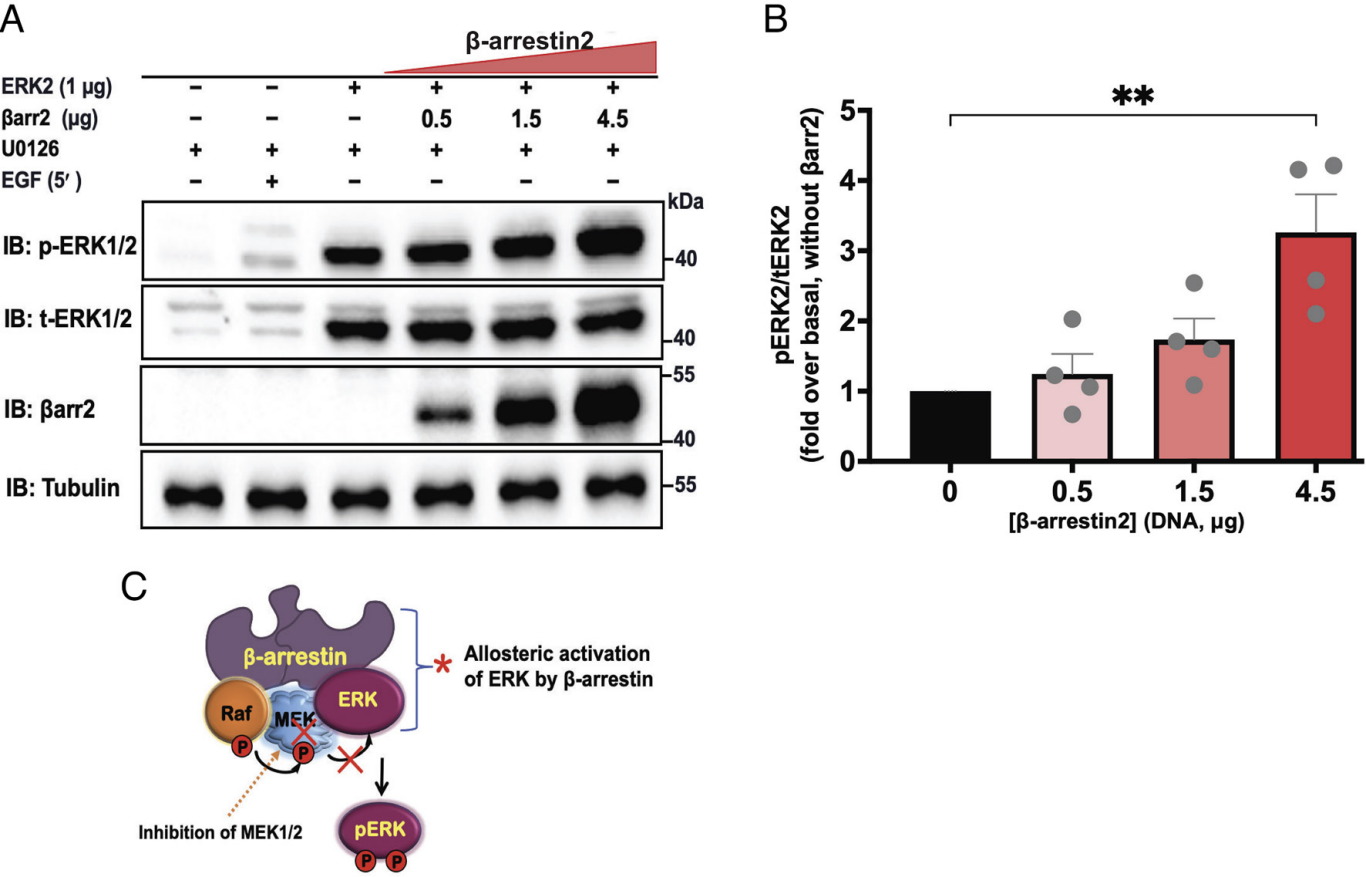
β-arrestin2 induces ERK2 activation in intact cells. (*A*) CRISPR/Cas9 βarr1/2 knockout HEK293 cells were transiently transfected with plasmids containing ERK2 and varying amounts of βarr2. After 48 h, cells were serum-starved and pretreated with vehicle (Dimethyl sulfoxide, DMSO) or 1 μM U0126 for 30 min. Lysates were immunoblotted for phospho-ERK2, total-ERK2, βarr2 (A1CT), and tubulin. (*B*) Quantification of MEK1/2- independent βarr-dependent ERK2 activation. Densitometry analysis of data from *A* is shown. Phospho-ERK signals were normalized to that of total-ERK2 and expressed as relative ratios to determine the fold response of βarr-mediated ERK2 activation compared to control. Data are means ± SEM of four independent experiments. Statistical test: one-way ANOVA with Bonferroni multiple comparison test, **P ≤ 0.01. (*C*) Cartoon showing a model of MEK-independent βarr mediated ERK Activation. The inhibition of MEK1/2 (chemically using U0126 herein) represents a tool to rule out the role of MEK1/2 in the allosteric activation of ERK1/2 by βarr1/2.

Next, we sought to investigate whether βarrs allosterically enhance ERK2 activity within intact cells. Given that βarr2 demonstrates a much stronger enhancement of ERK activity in vitro (particularly on MBP phosphorylation), we performed our investigation using this isoform. Accordingly, we transfected CRISPR/Cas9 βarr1/2 knockout HEK293 cells with expression plasmids encoding ERK2 and increasing amounts of βarr2 (Fig. 7). We utilized an upstream kinase MEK1/2 inhibitor, U0126 (42), to ensure that ERK2 phosphorylation signals result primarily from the allosteric effect of βarr rather than its scaffolding function (*SI Appendix*, Fig. S5). We initially confirmed that the MEK1/2 inhibitor effectively suppresses EGF-stimulated, MEK1/2-dependent ERK phosphorylation. Analysis of in-cell ERK2 kinase activity results (Fig. 7) showed that βarr2 induces ERK activation in a dose-dependent manner, with maximal activation achieved over threefold. These results using intact cells recapitulate those obtained from the in vitro kinase assay and further indicate that βarrs allosterically induce ERK2 activation. Collectively, our results strongly support the model that βarrs and GPCR–βarr complexes can allosterically regulate ERK activity towards downstream substrates.

## Discussion

βarrs are multivalent adaptor proteins that have been increasingly appreciated over the past two decades as playing broader roles in signal transduction by regulating a multitude of signaling pathways (1, 4, 5, 27). βarrs are necessary for receptor internalization and concomitantly initiate formation of GPCR–βarr multiprotein signalosomes, thereby acting as passive scaffolds that enhance receptor signaling efficiency (4, 12, 20, 21, 27). Among these, the assembly of RAF–MEK–ERK MAPK cascade components that leads to ERK1/2 activation represents one of the earliest and best-characterized examples of βarr scaffolding downstream of GPCR activation (12, 15, 21, 22, 43, 44). Surprisingly, in contrast to this classical model of βarrs acting simply as “passive” scaffolds, recent studies have shown that they can also enhance Src and c-Raf kinase activities via an allosteric mechanism (29–31). In the case of MAPK activation, however, it was not previously established whether βarr1/2 similarly allosterically stimulates ERK activity, as is the case for the analogous activity in yeast homologs (17, 32, 33).

Herein, we show that βarrs and their active states (i.e., bound to phosphopeptide or phosphorylated receptor) allosterically enhance ERK2 autophosphorylation and phosphorylation of downstream substrate by dually phosphorylated ERK. This regulation of ERK2 activities is the result of βarrs acting as allosteric modulators exerting their effects by binding ERK2. We demonstrated these physical interactions between ERK2 and βarrs using pulldown and coimmunoprecipitation approaches. Quantitative measurement of kinase activity revealed that both βarr1 and 2 enhance the ERK autophosphorylation rate more than 10-fold. Moreover, receptor-mediated activation of βarrs further enhanced the ERK2 autophosphorylation rate. We conclude that βarr binding to ERK allosterically promotes robust enhancement of its autophosphorylation activity, both in their basal states and more robustly in their active states.

We also identified the site at which ERK autophosphorylation activity is allosterically modulated by βarrs. Our data indicate that binding of βarrs to ERK2 induces its kinase activity by promoting phosphorylation of the ERK2 activation loop residue, Tyr^185^. Moreover, we demonstrated that this allosteric effect of βarrs on ERK2 Tyr-autophosphorylation occurs intramolecularly in a *cis* mechanism. Interestingly, we also found that the monophosphorylated ERK2-pY form of ERK2 exhibits moderately enhanced kinase activity towards MBP compared to its unphosphorylated form. This is consistent with the previously proposed idea that Tyr^185^ phosphorylation in ERK2 and similar monophosphorylation events in other kinases may serve as steps to establish a partially active conformation primed for subsequent full activation (13, 19, 32). Previous work has proposed that partial activity of ERK monophosphorylated on either activation loop residue (Thr or Tyr) may be linked to distinct biological roles or graded signal function activities (13, 14, 19). We found that βarrs and their active states also enhance the kinase activity of dually phosphorylated ERK2-pYpT towards MBP. Interestingly, our data revealed that βarr2 active forms exert a more substantial allosteric effect on ERK2-pTpY activity compared with the equivalent active form of βarr1. βarr2, in general, is reported to have a greater affinity for active phosphorylated receptors than βarr1 (34, 45). Thus, it is tempting to predict that the differential engagement of βarr isoforms with receptors may contribute to their distinct ERK2 activity. For example, βarr2 may stabilize a unique conformation that facilitates distinct allosteric regulation of ERK activity. The molecular details and physiological outcomes of such isoform-specific differentially allosteric activation of ERK1/2 warrant further study. Importantly, in further support of our in vitro results with purified components, we provide evidence that ERK2 allosteric regulation governed by βarr2 can also be recapitulated within intact cells. ERK2 activation occurs in a dose-dependent and MEK1/2-independent manner, arguing that such βarr-dependent activation of ERK2 may be a physiologically relevant process. It is worth noting, however, that in physiological settings, alteration in the location and expression (*SI Appendix*, Fig. S6) of the regulatory proteins βarrs and ERK in various tissues may differentially influence the activation output. Taken together, these data strengthen our model that ERK2 can draw upon its allosteric interaction with βarr to increase its kinase activity, suggesting an alternative mechanism with distinct signaling outcomes compared to the ERK1/2 activation signal from upstream parallel MAP kinase cascades.

There are several notable Ser/Thr kinases that undergo activation loop Tyr-autophosphorylation via a *cis* intramolecular mechanism, including Fus3, p38α, Raf, and GSK-3β (46–48). All these kinases undergo allosteric activity regulation following the recruitment of their protein-binding partners. Interestingly, the allosteric activation of ERK2 by βarrs closely resembles the activation of Fus3, a yeast homolog of ERK, by the Ste5 scaffold in the yeast mating pheromone response MAPK pathway (Ste11–Ste7–Fus3) (32, 33). Similarly, mammalian MAPK p38α has also been shown to be allosterically activated by binding its scaffold, transforming growth factor-β-activated kinase 1 (TAK1)-binding protein 1 (TAB1) (47, 49). Indeed, there seems to be a striking functional parallelism between the different allosteric regulator scaffold-kinase pairs. In many of these, the scaffold is itself phosphorylated by the activated kinase. Maximally active Fus3 is known to phosphorylate Ste5 (32, 33). Similarly, p38α phosphorylates TAB1 (49), and ERK phosphorylates βarrs (41); as noted above, we were careful to ensure that this did not confound our ERK2 autophosphorylation measurement. Such a mechanism may represent a general regulatory feedback mechanism for terminating or reducing the allosteric activation process. In addition, scaffold-mediated activation of a kinase is also known to govern many aspects of downstream cellular physiology; for example, TAB1-induced p38α autophosphorylation has been implicated in myocardial ischemia, T cell senescence, and skin and endothelial inflammation triggered by receptor ligands (50). ERK1/2 is likewise known to play diverse physiological roles activated by a myriad of mechanisms and, in turn, propagate signaling events via phosphorylation of over 100 substrates (13, 14). Although the exact mechanism of βarr-induced ERK2 activation is unknown, it is possible that βarr binding allosterically induces a conformational change in the ERK2 activation loop that mediates priming for further activation in a similar manner as observed for the Fus3/Ste5 and p38a/TAB1 complexes (32, 47). As postulated above, our results suggest that βarr allosterically enhances ERK2 autoactivation by converting it from an intrinsically inactive to a partially active state. Current studies are directed towards elucidating the mechanism and physiological implications of such allosteric ERK activation by βarrs.

In summary, our findings demonstrate that βarrs do not function simply as passive scaffolds in assembling the MAPK effector pathway but rather also play an active role as “catalytic” scaffolds in allosterically unlocking ERK activity to regulate MAPK signaling. Our work thus elucidates a previously unappreciated mechanism, through which βarrs, coupled to active phosphorylated receptors, modulate the activity of downstream effectors. The relative contributions of scaffolding and allosteric activation roles of βarrs in enhancing ERK activation by GPCRs in vivo remain to be unraveled. However, these findings highlight the vital role that βarrs play as true transducers of GPCR signals. Moreover, taken together with other recent findings of allosteric modulation of c-Src and c-Raf kinase activity by βarrs, these findings support the notion that βarrs may generally function dually as both scaffolds and allosteric regulators to differentially modulate the activity of downstream enzymes.

## Materials and Methods

Details of the *Materials and Methods* used in this paper, including *Cell Culture, Transfection, and Materials; Expression and Purification of Chimeric β_2_-adrenergic Receptor; Recombinant Protein Expression and Purification; Coimmunoprecipitation and Immunoblotting; Pull-Down of ERK2–β-arrestin1/2 Complexes; Mass Spectrometry-Based Detection of ERK2 Phosphosite; In Vitro ERK2 Autophosphorylation Kinase Assay and Immunoblotting; In Vitro [*γ-32P*]*ATP ERK2 Kinase Assay; ERK2 Kinetics In Vitro Kinase Assay; In Cell ERK Activation Assay; Quantification of Protein Expression Levels in Cells;* and Statistical Analysis* are provided in *SI Appendix*, *Materials and Methods*.

## Supplementary Material

Appendix 01 (PDF)

## Data Availability

All study data are included in the article and/or *SI Appendix*.
