# Supplementary material for: Signal transduction at GPCRs: Allosteric activation of the ERK MAPK by β-arrestin
Source: Proc Natl Acad Sci U S A. 2023 Oct 16;120(43):e2303794120. doi: 10.1073/pnas.2303794120 (PMC10614829; doi:10.1073/pnas.2303794120)
Supplement: Supplementary file 1 — Appendix 01 (PDF) [file pnas.2303794120.sapp.pdf]

PNAS

Supporting Information for

# Signal Transduction at GPCRs: Allosteric Activation of the ERK MAPK by β-arrestin

Alem W. Kahsai[a], Kunal S. Shah[a,b], Paul J. Shim[a,c], Mason A. Lee[a], Bowie N. Shreiber[a], Allison M. Schwalb[a,b], Xingdong Zhang[a], Henry Y. Kwon[a,d], Li-Yin Huang[a], Erik J. Soderblom[e,f], Seungkirl Ahn[a] , and Robert J. Lefkowitz[a,g,h,i,1,]

[a]Department of Medicine and [b]Duke University School of Medicine, Duke University Medical Center, Durham, NC 27710

[c]The University of Arizona, Department of Medicine, College of Medicine, Phoenix, AZ 85004

[d]Henry Ford Hospital, General Surgery Residency Program, Detroit, MI 48202

[e]Department of Cell Biology, [f]Duke Center for Genomic and Computational Biology, [g]Department of Biochemistry, and [h]Department of Chemistry, Duke University Medical Center, Durham, NC 27710

[i]HHMI, Duke University Medical Center, Durham, NC 27710

[1]To whom correspondence may be addressed. Email: lefko001@receptor-biol.duke.edu.

**This PDF file includes:**

Supporting text: Materials and Methods

Figures: S1 to S6

SI References

## Supporting Text

## Materials and Methods

### Cell Culture, Transfection, and Materials.

Human embryonic kidney 293 (HEK293) and HEK293 CRISPR/Cas9 βarr1/2 knockout (KO) cells were cultured in minimum Eagle's media (MEM) supplemented with 2 mM L-glutamine, penicillin-streptomycin, and 10% fetal bovine serum, and cells were maintained in an incubator with 5% $CO_2$ at 37°C. For cell starvation, serum-free medium supplemented with 0.1% BSA, 10 mM HEPES, and 1% penicillin–streptomycin was used. Transient transfections were performed using Lipofectamine 3000 (ThermoFisher Scientific # L3000008) and FuGENE® 6 (Promega) according to the manufacturer's instructions. Monoclonal anti-FLAG M2-HRP (A8592), monoclonal anti-ERK1/2 (ABS44) and monoclonal anti-α-Tubulin (T5168) antibodies were obtained from Sigma. Complete protease (11836153001) and PhosStop phosphatase inhibitor (4906837001) cocktail tablets were purchased from Roche (Basel, Switzerland). The MEK1/2 inhibitor (U0126)(1) was purchased from Selleckchem (Catalog No.S1102). Monoclonal anti-hemagglutinin (HA) affinity agarose was obtained from Thermofisher (Princeton, NJ). Anti phospho–p44/42 MAPK (ERK1/2) ($Thr^{202}$/$Tyr^{204}$) (9101L) and anti phospho–p44/42 MAPK (ERK1) ($Tyr^{204}$)/(ERK2) ($Tyr^{187}$) (5726S) (2) were purchased from Cell Signaling Technology. Anti-HA tag HRP (ab173826), Anti-6X His tag (ab18184), and Phospho pT-44/42 MAPK (ERK1) ($pThr^{202}$)/(ERK2) ($pThr^{185}$) (ab222493) (3) antibodies were purchased from Abcam (Waltham, MA). $V_2$Rpp and $V_2$Rnp, phosphorylated (shorter version with 6-distal phosphates) and non-phosphorylated peptides, respectively, were synthesized by the Tufts University analytical core facility. Isoproterenol (Sigma I2760-1G) and BI-167107 were prepared as 100 mM stocks in DMSO. Agonist stimulations were performed at 37°C in serum-free media as described in the figure legends.

### Expression and Purification of Chimeric $\beta_2$-Adrenergic Receptor.

The N-terminal FLAG-tagged with tobacco etch virus (TEV) cleavage site to T4 Lysozyme fusion-$\beta_2V_2$R chimeric receptor was co-expressed with or without GRK2-CAAX in Sf9 insect cells using the Baculovirus Expression System as described previously (4-7). At 66 h post-infection, cells were harvested either immediately or after stimulation with

isoproterenol to generate T4L fusion phosphorylated-$\beta_2V_2R$ here in the study named as p$\beta_2V_2R$ (4, 6, 8). Next, cells were lysed using a lysis buffer (10 mM Tris, pH 7.4, benzamidine [10 mg/mL], leupeptin [10 mg/mL]) and subsequently solubilized in an n-Dodecyl-β-D-maltoside (DDM; Anatrace)-containing solubilization buffer [1.0% DDM, 500 mM NaCl, 20 mM Tris, pH 7.4, benzamidine (10 mg/mL), leupeptin (10 mg/mL), PMSF (200 µmol)]. Functional $\beta_2V_2R$ and $\beta_2V_2R$ were obtained in 0.01% (w/v) maltose neopentyl glycol (MNG) containing 20 mM HEPES, pH 7.5, 100 mM NaCl buffer using a three-step affinity-chromatographic procedure: first involving an M1 anti-FLAG-antibody affinity column, followed by an alprenolol-ligand affinity column and lastly an additional M1 anti-FLAG antibody-affinity column. For some downstream purposes receptor samples were incubated with TEV protease (Sigma; T4455) to cleave off the N-terminal FLAG-tag. All receptor samples were further purified by size-exclusion chromatography (SEC) using a Superdex 200 Increase 10/300 GL column attached to an ÄKTA fast protein liquid chromatography (FPLC) system (GE Healthcare). Receptor samples were flash-frozen in small aliquots after adding 15% glycerol and stored at −80°C until further usage.

**Recombinant Protein Expression and Purification.**

The expression and purification of βarrs have been described previously (4, 6, 9). Briefly, *E. coli* Bl21 Rosetta (DE3) pLysS cells (Millipore sigma: 70954) carrying pGEX4T1-*Rattus norvegicus* βarr1 or 2 construct, with their C-terminal truncated (at amino acid 393 or at 394, respectively), were cultured in Terrific Broth (Teknova, Hollister, CA) medium at 37°C. After $OD_{600}$ reached 0.6–0.8, the cells were induced with 0.1 mM isopropyl-β-D-thiogalactopyranoside (IPTG) at 18°C overnight. Bacteria were collected by centrifugation (4000 RPM). Cell pellets were resuspended in lysis buffer [20 mM HEPES, pH 8, 150 mM NaCl, 10% Glycerol, 1mM EDTA, 0.2 mM dithiothreitol (DTT), 1 mM phenylmethyl sulphonyl fluoride, and 1mM benzamidine] at 4°C for 1 hr. Cell suspensions were sonicated, centrifuged (14,000 RPM, 30 min, 4°C) and then loaded onto pre-equilibrated Glutathione-agarose resin (GoldBio). After 3 h of binding at 4°C, the beads were washed, and thrombin was allowed to incubate with the beads for overnight cleavage. Finally, proteins were subjected to anion exchange chromatography followed by SEC using a Superdex 200 Increase 10/300 GL column on ÄKTA FPLC system (GE Healthcare).

Eluted fractions were analyzed by SDS–PAGE, and fractions of interest were pooled, concentrated on 30 kDa MWCO Amicon Ultra-15 Centrifugal Filter devices, flash-frozen in liquid nitrogen, and stored in aliquots at −80°C until further usage. WT-ERK2 and its GST-tagged kinase-dead (KD) variant (GST–ERK2 K52A) were purified similarly using codon optimized wild-type rat ERK2 gene synthesized (Genscript) and sub-cloned into pGEX4T1 vector. Dually phosphorylated ERK2 (ERK2-pTpY; phosphorylated at $Thr^{183}$ and $Tyr^{185}$) was obtained via *in vitro* kinase assay incubation of wt-ERK2 with GST-tagged active MEK1 (sigma: M8822-10UG) at 37 °C for 1 h. Monophosphorylated ERK2-pY ($Tyr^{185}$) was prepared from wt-ERK2 by an *in vitro* kinase assay using ATP as a phosphate source in kinase buffer, as described below. The phosphorylation state of each ERK2 form was assessed by western blotting using phospho-specific (ERK2-pY, ERK2-pT, and ERK2-pTpY) and total ERK2 antibodies. 6xHis-tagged Nb32 was purified as described previously (6, 10).

**Coimmunoprecipitation and Immunoblotting.**

Immunoprecipitation experiments were performed following transient transfection of AI-HEK293 CRISPR/Cas9 βarr1/2 knockout cells (11) in 150-mm dishes, as previously described(12, 13). Cells were co-transfected with expression plasmids encoding $\beta_2V_2R$ and ERK2, as well as with either empty vector or plasmids encoding HA-tagged βarr1 or 2, as indicated (Lipofectamine 3000; Thermo Fisher Scientific). After 48 h, cells were serum-starved (overnight) and then stimulated with agonist isoproterenol (10 μM) for 7 min. Cells were then placed on ice, and dithiobis(succinimidyl propionate) (DSP) cross-linking reagent (1 mM; Thermo Fisher Scientific) was added dropwise. The cell lysates in (10 mM Tris, pH 7.4, 100 mM NaCl, 1 mM EDTA, 1 mM EGTA, 1 mM NaF, 20 mM $Na_4P_2O_7$, 2 mM $Na_3VO_4$, 0.5% Triton X-100, 10% glycerol, 0.05% deoxycholate, 1 mM PMSF, 10 μg/ml leupeptin, and 10 μg/ml aprotinin) were subjected to coimmunoprecipitation with anti-HA antibody affinity gel (40 μL) and incubated for 3 hrs at 4 °C. The immuno-protein complexes were precipitated by centrifugation, washed three times and resuspended in 80 μl of 2× Laemmli sample buffer. The protein complexes were then subjected to SDS-PAGE, immunoblotting, and chemiluminescent imaging using ChemiDoc-XRS charge-coupled device camera system (Bio-Rad Laboratories).

βarr1/2 and ERK2 immune complexes were detected by immunoblotting using an anti-phospho-ERK1/2 antibody (anti-ERK2-pT183/pY185 for rat species), total ERK2, or anti-HA antibodies for βarr1/2. 50-µL aliquots whole cell lysates (TCL) of each sample were mixed with an equal volume of 2× Laemmli sample buffer and resolved by SDS-PAGE to confirm equal loading by immunoblotting with antibodies against tubulin, $\beta_2V_2R$ (anti-FLAG HRP), t-ERK, and βarr1/2 (A1CT). For immunoblot-based pull-down and kinase assay input analysis involving other baculovirus–insect and bacterially expressed proteins, similar immunoblotting analysis was performed (for $p\beta_2V_2R$ anti-FLAG-HRP was used to probe N-terminally FLAG-tag intact T4L-$p\beta_2$V2R and anti-6×His-HRP for Nb32).

**Pull-down of ERK2–β-arrestin1/2 Complexes.**

Pull-down of 3xFLAG-ERK2 was performed using monoclonal M2 anti-Flag affinity agarose. Beads were washed 3 times with binding buffer [20 mM HEPES, pH 7.5, 125 mm NaCl and 0.5% glycerol]. Purified 3xFLAG-tagged ERK2 was incubated with βarr1 or 2 alone (5 µM) or each in their active state forms bound to $V_2$Rpp (50 µM) or $p\beta_2V_2R$ (10 µM) together with the active βarr-stabilizing nanobody (Nb32, 20 µM) for 30 min initially on ice. The reaction mixtures were added to 30 µL of anti-FLAG M2 agarose beads (Sigma). After further incubation for 1 h at room temperature, the beads were washed three times with binding buffer and eluted with binding buffer containing 0.5 mg/mL FLAG peptide (GenScript) and 10 mM EDTA. Finally, 3xFLAG-ERK2 and associated βarr1 or 2 in the complex of 3xFLAG-ERK2–βarr1 or 2 were analyzed by western blotting using the total ERK1/2 antibody (Millipore-Sigma, ABS 44; 1:10,000) and anti-βarr1 A1CT antibody that recognize both βarr1 and 2 (1:3,500).

**Mass Spectrometry-Based Detection of ERK2 Phosphosite.**

Purified recombinant rat wild-type ERK2 protein (2.4 µM) was incubated either alone or in the presence of active βarr2 (40 µM) bound to $V_2$Rpp (400 µM) in kinase buffer (25 mM Tris-HCl, pH 7.5, 100 mM NaCl, 5% glycerol, 10 mM $MgCl_2$, 5 µM TCEP, protease inhibitors, and phosphatase inhibitors). The autophosphorylation reaction was initiated with the addition of 2 mM ATP. ERK2 alone without ATP was used as a negative control. The autophosphorylation reactions proceeded for 60 min at 30 °C and were terminated

by flash freezing in liquid nitrogen and were stored in a − 80°C deep freezer until LC–MS/MS analysis. To perform in-solution trypsin digestion, samples were supplemented with 5% SDS, reduced with 5 mM DTT for 15 min at 80°C, alkylated with 20 mM iodoacetamide for 30 min at room temperature, then supplemented with a final concentration of 1.2% phosphoric acid and 609 µL of S-Trap (Protifi, Fairport, NY) binding buffer (90% MeOH/100mM TEAB). Proteins were trapped on the S-Trap micro cartridge, digested using 20 ng/µL sequencing grade trypsin (Promega) for 1 h at 47°C, and eluted using 50 mM TEAB, followed by 0.2% formic acid (FA), and lastly using 50% acetonitrile (ACN)/0.2% FA. All samples were then lyophilized to dryness. Each sample was subjected to chromatographic separation on a Waters MClass UPLC equipped with a 1.7 µm HSS T3 $C_{18}$ 75 µm I.D. X 250 mm reversed-phase column (NanoFlow data). The mobile phase consisted of (A) 0.1% FA in water and (B) 0.1% FA in ACN.  3 µL was injected and peptides were trapped for 3 min on a 5 µm Symmetry $C_{18}$ 180 µm I.D. X 20 mm column at 5 µl/min in 99.9% A. The analytical column was then switched in-line and a linear elution gradient of 5% B to 40% B was performed over 90 min at 400 nL/min. The analytical column was connected to a Fusion Lumos mass spectrometer (Thermo) through an electrospray interface operating in a data-dependent mode of acquisition. The instrument was set to acquire a precursor MS scan from *m/z* 375-1500 at R=120,000 (target AGC 2e5, max IT 50 ms) with MS/MS spectra acquired in the ion trap (target AGC 1e4, max IT 50 ms). For all experiments, HCD energy settings were 30% and a 20 s dynamic exclusion was employed for previously fragmented precursor ions.

Raw LC-MS/MS data files were processed in Proteome Discoverer 2.5 (Thermo Scientific) and then submitted to independent Mascot database searches against a *Rattus norvegicus* protein database containing both forward and reverse entries of each protein. Search tolerances were 2 ppm for precursor ions and 0.8 Da for-product ions using trypsin specificity with up to two missed cleavages. Carbamidomethylation (+57.0214 Da on C) was set as a fixed modification, whereas oxidation (+15.9949 Da on M) and phosphorylation (+79.9663 Da on STY) were considered dynamic mass modifications. Phosphorylated peptide abundances were determined using their intensities extracted from ion chromatograms for each reaction condition within Protein Discoverer. The

phosphorylation status and peptide abundance of the regulatory phosphorylation sites $Thr^{183}$ and $Tyr^{185}$ with and without active βarr2 were compared and intensities were normalized to the control reaction of ERK2 alone with ATP (treated as 1).

***In Vitro* ERK2 Autophosphorylation Kinase Assay and Immunoblotting.**
The ERK2 autophosphorylation kinase assay (steady state format; as assessed by endpoint immunoblotting) mixtures contained kinase buffer (20 mm HEPES, pH 7.5, 10 mm $MgCl_2$, 5% Glycerol, 5 µM TCEP, complete protease, and PhosStop phosphatase inhibitor cocktails), 0.5 mM of ATP, and 3xFLAG-tagged ERK2 (30 nM or as indicated in the figure legends). The reactions contained ERK2 in the absence or presence of 1 µM βarr1 or 2 or each in their active state forms bound to either $V_2Rpp$ (10 µM) or BI-167107(10 µM)-$p\beta_2V_2R$ (2.5 µM) together with an active βarr-stabilizing nanobody (Nb32, 4 µM). Kinase reactions were initiated with 0.5 mM ATP. After incubation at 30 °C for 30 min, reactions were terminated by the addition of equal amounts of 2X Laemmli Sample Buffer (Bio-Rad) and subsequent flash-freezing in liquid nitrogen. The samples were then resolved by SDS-PAGE (4-to-20% gradient) and transferred to nitrocellulose membranes. Phosphorylated ERK1/2 and total ERK1/2 were detected by immunoblotting with rabbit polyclonal anti-phospho-p44/42 MAPK (1:3000; Cell Signaling) antibody and for total ERK2 using HRP-conjugated anti-FLAG M2 (Sigma, 1:4000) or anti-MAPK1/2 (1:10,000; Millipore-Sigma) antibody. The phosphorylation state of the monophosphorylated forms of ERK2 was assessed using phosphorylation site-specific antibodies that recognize $pT^{183}$ (ab222493; Abcam) or $pY^{185}$ (5726S; Cell Signaling Technology) within the regulatory TEY activation loop. Assays to determine the autophosphorylation mechanism were performed similarly by incubating wild-type ERK2 (30 nM) with a GST-tagged kinase-dead (KD) ERK2 mutant (GST-ERK2-K52A; 45 nM), to be able to distinguish them by their size. Protein bands on nitrocellulose membrane were detected with SuperSignal West Pico enhanced chemiluminescent substrate (Thermo Fisher) and captured using a ChemiDoc-XRS charge-coupled device camera system (Bio-Rad Laboratories). Bands were quantified by densitometry using Image Lab (Bio-Rad, Hercules, CA), and GraphPad Prism software was used for data analyses.

### *In Vitro* [γ-$^{32}$P]ATP ERK2 Kinase Assay.

The kinase activity of dually phosphorylated ERK2-pTpY ($Tyr^{185}$ and $Thr^{183}$) towards MBP (MilliporeSigma, bovine) in the absence or presence of βarr1/2 or their active states was assayed by an *in vitro* kinase assay in which $^{32}$P from [γ-$^{32}$P]ATP incorporated into myelin basic protein (MBP) was directly measured by autoradiography. ERK2-pTpY (15 nM) was incubated with [γ-$^{32}$P]ATP (10 μM, 8000 cpm/pmol) and MBP (100 nM) at 30 °C for 60 min in a kinase buffer (20 mm HEPES, pH 7.5, 10 mm $MgCl_2$, 5% Glycerol, 5 μM TCEP, complete mini protease, and PhosStop phosphatase inhibitor cocktails). The reactions contained ppERK2 in the absence or presence of 300 nM βarr1 or 2 or each in their active state forms bound to either $V_2$Rpp (1.5 μM) or BI-167107(5 μM)-p$\beta_2V_2$R (1.5 μM) together with an active βarr-stabilizing nanobody (Nb32, 1.2 μM). The kinase reactions were terminated by the addition of equal amounts 2X Laemmli Sample Buffer (Bio-Rad). The kinase activity of mono-phosphorylated ERK2-pY (at $Tyr^{185}$) was also assessed similarly using MBP as a substrate and [γ-$^{32}$P]ATP as a phosphate source. Dually phosphorylated ERK2-pTpY (at $pT^{183}$ and $pY^{185}$) and non-phosphorylated ERK2 forms were used as positive and negative controls, respectively. In both experimental settings, the incorporation of $^{32}$P into MBP was analyzed by SDS-PAGE, followed by autoradiography using a PhosphorImager and a Molecular Typhoon Phosphorimager (GE Healthcare).

### ERK2 Kinetics *In Vitro* Kinase Assay.

The allosteric effect of βarrs on ERK2 autophosphorylation activity was determined using a fluorescent-based real-time *in vitro* kinase assay format according to the manufacturer's instructions (BioVision, Inc.). The assay monitors ATP consumption by a kinase (herein ERK2) and relies on measuring the formation of ADP detected as a fluorescence readout using an ADP-specific sensor. The ERK2 autophosphorylation kinase reaction was performed in kinase buffer (20 mm HEPES, pH 7.5, 10 mm MgCl2, 5% Glycerol, 5 μM TCEP, complete protease, and PhosStop phosphatase inhibitor cocktails) using wt-ERK2 (15 nM) alone or in the presence of 300 nM βarr1 or 2 or each in their active state forms bound to either $V_2$Rpp (3 μM) or 1 μM p$\beta_2V_2$R (20 μM isoproterenol at) together with Nb32 (1.2 μM) in 96-well, half-volume, flat-bottom, opaque black plate (Corning, Tewksbury, MA). Following the addition of 75 μM ATP and incubation (10 min, RT), changes in

fluorescence were measured kinetically at an excitation wavelength of 540 nm and emission reading at 590 nm on a CLARIOstar microplate reader (BMG Labtech, Germany) at 30°C. Each experiment included control wells containing βarr 1 or 2 (with ATP) without ERK2 and another control well containing ERK2 alone without ATP. There was no signal measured in the negative control of ERK2 alone without ATP, while the second control reaction containing βarr 1 or 2 with ATP (lacking ERK2) was used for background correction. The RFUs at each time point of this control were subtracted from the RFUs of the corresponding time point of the experimental sample readings. Corrected intensity values (normalized, RFUs) were plotted as a function of time. Initial rates were determined from curve fitting of the linear phase portion of the reactions using linear regression in GraphPad Prism (GraphPad Software, Inc, La Jolla, CA). The kinetics of ERK autophosphorylation are presented as fold-enhancement of initial rates from each reaction profile relative to vehicle control (ERK2 alone treated as 1-fold).

The ability of βarrs to modulate the kinase activity of dually phosphorylated ERK2-pTpY toward MBP was also assessed similarly using this real-time kinase assay. The kinase activity of ERK2-pTpY (7.5 nM) to phosphorylate MBP (100 nM) was measured in the absence or presence of 200 nM βarr1 or 2 or each in their active state forms (bound to either $V_2$Rpp [1 μM] or isoproterenol-occupied-p$\beta_2V_2$R [1 μM], together with active βarr stabilizing nanobody, Nb32 [0.8 μM]). Following the addition of ATP (50 μM) and incubation (10 min, RT), changes in fluorescence were measured kinetically at an excitation wavelength of 540 nm and emission reading at 590 nm on a CLARIOstar microplate reader (BMG Labtech, Germany) at 30°C. Given that βarr1 and 2 are known substrates for ERK (14), each experiment was designed to include two control reaction wells in the presence of ATP to prevent confounding from any such phosphorylation. The first contained ERK2-pTpY but no MBP or βarr1 or 2 and was used to subtract any contribution due to ERK2-pTpY autophosphorylation. The second contained ERK2-pTpY and βarr1/2 but no MBP (which values were negligible under these assay conditions) and was subtracted to remove any contribution from βarr1/2 phosphorylation. Corrected intensity values (normalized, RFUs) were plotted as a function of time. Initial rates were determined from curve fitting of the linear phase portion of the reactions using linear

regression in GraphPad Prism (GraphPad Software, Inc, La Jolla, CA). The kinetics of ERK2-pTpY activity toward MBP phosphorylation are presented as fold-enhancement of initial rates from each reaction profile relative to vehicle control (absence of βarr treated as 1-fold).

**In Cell ERK Activation Assay**

HEK293 CRISPR βarr1/2 KO cells (15), generously provided by the Laporte Laboratory (McGill University, Montreal, Quebec, Canada) were seeded on 6-well plates in MEM supplemented with 10% FBS, 1% penicillin-streptomycin at 37°C. After 24 h, cells were transfected simultaneously with empty plasmid pcDNA3.1 vector, expression plasmid for pCMV5-ERK2 (1 µg), and pcDNA3-βarr2-HA (0, 0.5, 1.5, 4.5 µg) in a dose-dependent manner. After 48 h, cells were starved for 3 h in serum-free media buffered with 10 mM HEPES pH 7.4. Cells were treated with either vehicle (DMSO) or 1 µM U0126 [2] for 30 min. EGF stimulation was performed at 0.1 ng/mL for 5 min. Cells were then lysed by direct addition of 2X SDS-sample buffer followed by sonication. Equal amounts of cellular extracts were separated on 4-20% SDS-PAGE gels and transferred to nitrocellulose membranes for immunoblotting. Phosphorylated ERK1/2, total ERK1/2, and βarrs were detected by immunoblotting using rabbit polyclonal anti-phospho-p44/42 MAPK (Cell Signaling, 1:2,500), anti-MAP kinase 1/2 (Upstate Technology Inc, 1:5,000), and anti-βarr1/2 (A1CT, 1:2,500) antibodies, respectively. Chemiluminescence was detected by SuperSignal West Pico reagent (Pierce) using Chemidoc Touch Imaging System (Bio-Rad), and densitometry analysis of immunoblots was performed on Image LabTM 6.0 software (Bio-Rad). To assess ERK activation, densitometry values of bands were normalized by dividing the intensity of phospho-ERK2 by total ERK2 and expressed as relative ratios to determine the fold response of βarr-mediated ERK2 activation compared to control. Statistical significance was assessed by one-way ANOVA with a Bonferroni multiple comparisons post-test.

**Quantification of Protein Expression Levels in Cells**

Total cell lysates were prepared from HEK293 and HEK293 βarrestin1/2 CRISPR KO (SL) cell lines. Whole cell lysate protein concentration was determined using a BCA protein

assay kit (Thermo Scientific™ Pierce™). Purified recombinant βarr 1 and 2 and ERK2 were used as protein standards to generate curves. Equal amounts of protein were separated by SDS/PAGE and immunoblotted with rabbit polyclonal anti-βarr antibody (A1CT) or anti-ERK1/2 antibody. Relative quantitation of βarr1/2 or ERK1/2 levels was obtained by densitometric immunoblot analysis using the established standard curves.

**Statistical Analysis.**

All statistical analyses were carried out using GraphPad Prism 9 (GraphPad Software, Inc, La Jolla, CA). Statistical significance was assessed by performing one-way analysis of variance (ANOVA) with Dunnett's or Tukey's post-hoc test. Two-way ANOVA with Bonferroni correction for post-hoc analysis was used for repeated measures or with an array of conditions. Differences with at least a *P*-value of $< 0.05$ were considered significant.

## SI Figures

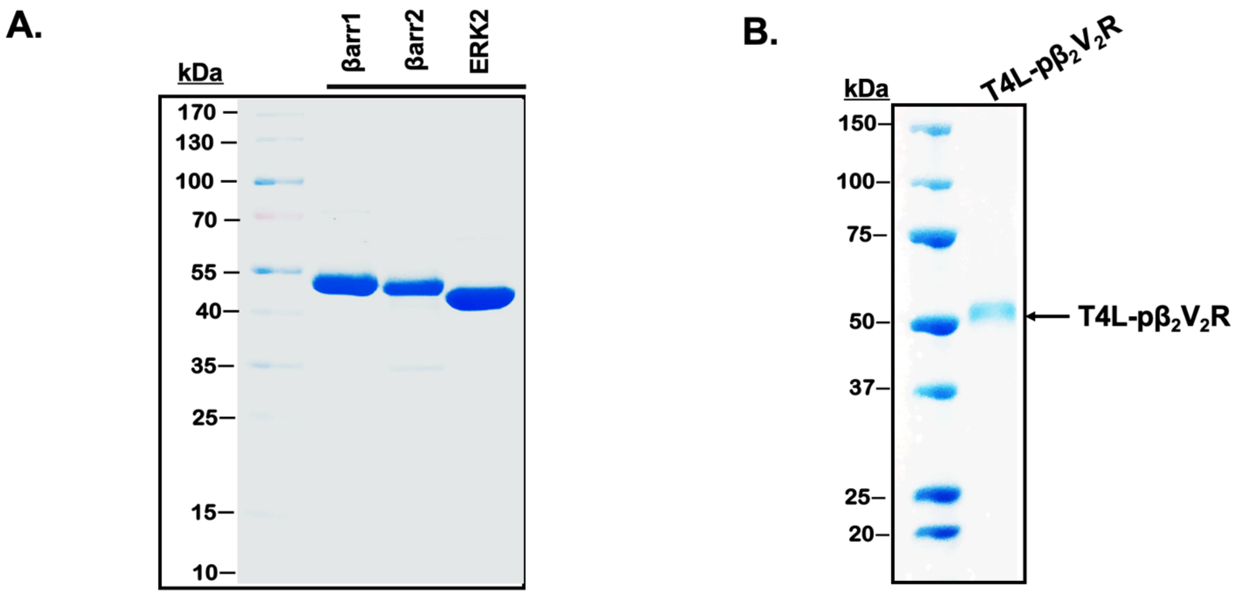


**Fig. S1. Biochemical Characterization of β-arrestin1/2, ERK2, and T4L-pβ$_2$V$_2$R Recombinant Proteins**. Size exclusion chromatography (SEC) purified, SDS–PAGE gel runs of βarr1/2 and ERK2 (panel ***A***), and detergent (MNG)-solubilized T4L-pβ$_2$V$_2$R (herein named pβ$_2$V2R) (panel ***B***), as visualized by Coomassie Brilliant blue staining. In panel A, lane 1, βarr1 (truncated form: 1-393); lane 2, βarr2 (truncated form: 1-394); and lane 3, ERK2. The molecular weight of protein bands estimation is shown and performed using PageRuler™ Prestained Protein Ladder (panel ***A***; Thermo Scientific) and Precision Plus Protein™ All Blue Standards (panel ***B***; Bio-Rad).

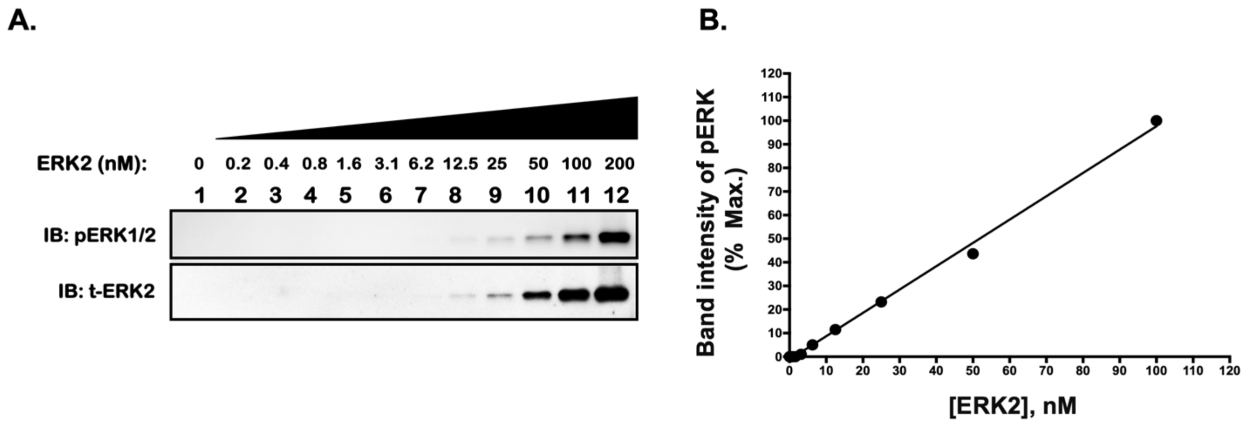


**Fig. S2.** ***In vitro*** **Autophosphorylation of ERK2. (*A*)** An *in vitro* kinase assay with a series of concentrations of 3xFLAG tagged ERK2 (0.2 nM – 200 nM) was performed at 30 ℃ in an endpoint immunoblotting analysis format for 30 min using ATP (500 μM). The autophosphorylation state of ERK2 was assessed by immunoblotting with an antibody against phospho-ERK1/2 (anti-ERK2-pT$^{183}$/pY$^{185}$ for rat species). Equal loading is shown for the unphosphorylated ERK2 using an antibody for total-ERK2 (anti-FLAG-HRP). **(*B*)** Quantification of ERK band phosphorylation intensities shows linear correlation with corresponding ERK2 concentrations, confirming the autophosphorylation of ERK2 is a single reaction event (slope = 0.9883 ± 0.0184; $R^2$ = 0.9967).

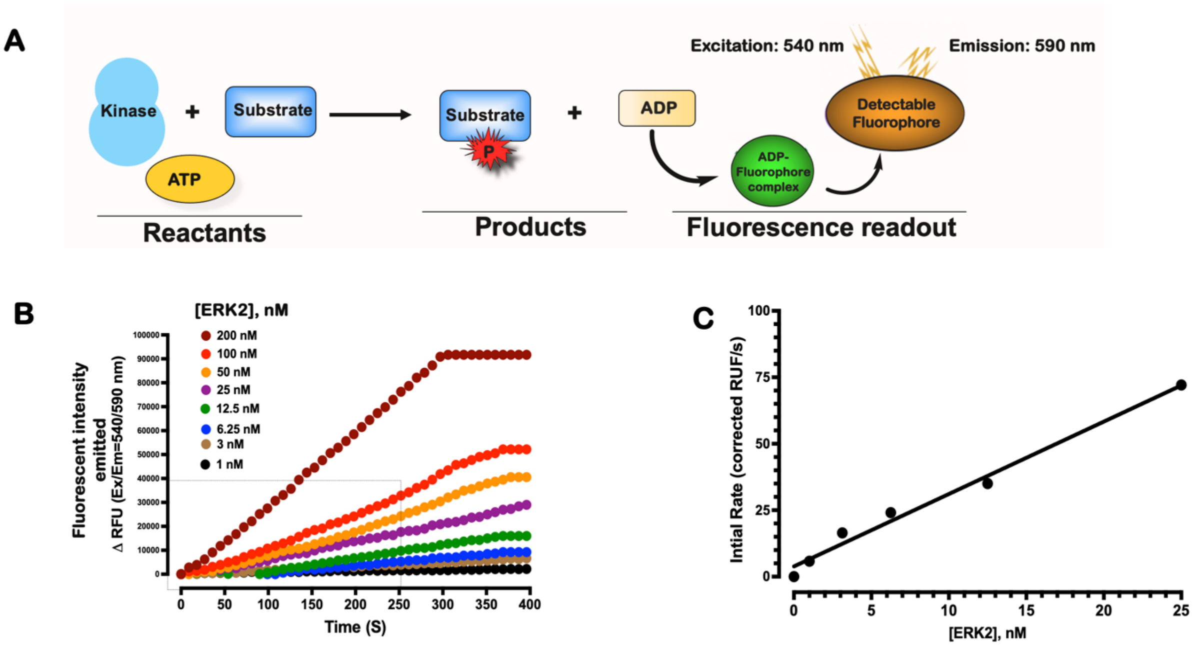


**Fig. S3. ERK2 Phosphorylates Itself as Assessed by Real-time Fluorescent-Based Monitoring of Kinase Activity. (*A*)** Schematic illustration of fluorescent-based real-time monitoring of ERK2 kinase activity. The assay monitors ATP consumption by a kinase (herein ERK2) and relies on measuring the formation of ADP detected as a fluorescence readout using an ADP-specific sensor. The fluorescence of the ADP-fluorophore complex is excited at 540 nm, and emission fluorescence is detected at 590 nm. **(*B*)** Time courses of ERK2 autophosphorylation showing assay validation performed using a series of increasing concentrations of ERK2 (1 nM – 200 nM) at 30 ℃, demonstrating its suitability for measuring real-time ERK activity in vitro. **(*C*)** Quantitative analysis of ERK2 autophosphorylation from *in vitro* kinase experiments shown in **(*B*).** The initial rate of reactions was calculated from linear fits as the initial slope of lines from each reaction profile (normalized relative fluorescent units, RFU as a function of time in sec) and was plotted as a function of ERK2 concentration (slope = 2.724 ± 0.1684; $R^2$ = 0.9849).

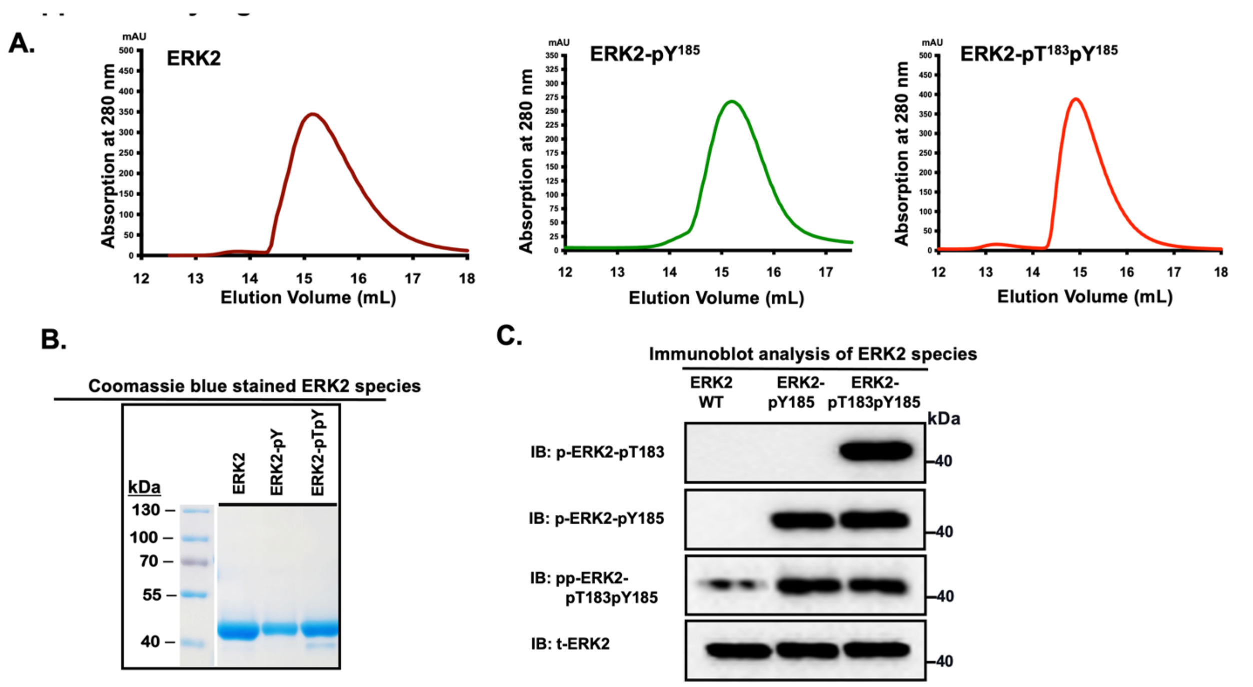


**Fig. S4. Biochemical Characterization of Phosphorylated and Unphosphorylated Forms of ERK2 (*A*)**. Elution profiles after size exclusion chromatography (SEC) through a Superdex-200 10/30 column are shown for the unphosphorylated, monophosphorylated on Tyr$^{185}$, and dually phosphorylated on Thr$^{183}$ and Tyr$^{185}$ forms of rat ERK2. (***B***) SEC-separated ERK2 forms were Coomassie blue stained after resolution on SDS–PAGE gel. ERK2-pY was prepared using autophosphorylation from WT-ERK2 by an *in vitro* kinase assay using ATP as a phosphate source in kinase buffer as described in Materials and Methods at 30 °C. ERK2-pTpY was prepared in an *in vitro* kinase assay using active human GST–MEK1 at 37 °C for 1 hr. Both were purified via SEC, as shown in ***A***. (***C***) The phosphorylation state of each ERK2 form was assessed by western blotting using phospho-specific (ERK2-pY, ERK2-pT, and ERK2-pTpY) and total ERK2 antibodies.

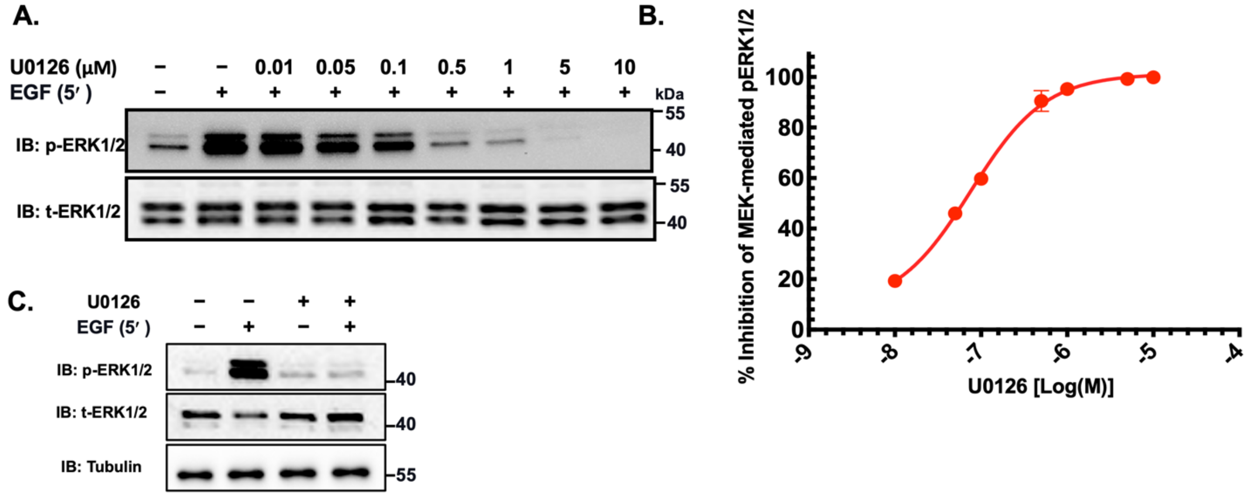


**Fig.S5. MEK1/2-Dependent EGFR-mediated ERK1/2 Phosphorylation Inhibition**. (***A***) Dose-dependent Effect of the MEK Inhibitor U0126 on EGFR-mediated ERK1/2 Phosphorylation**.** The MEK inhibitor U0126 inhibited EGF (0.1 ng/mL) stimulated phosphorylation of ERK1/2 in a concentration-dependent manner. Results displayed represent typical immunoblots obtained using phospho-ERK1/2 antibody. (***B***) Phospho-ERK1/2 levels were determined by densitometric analysis of the immunoblots. The resulting phospho-ERK levels are plotted as percent of maximum (vehicle). Data represent the mean ± SEM of duplicate experiments. (***C***) Immunoblot analysis of ERK phosphorylation demonstrates that the MEK inhibitor U0126 (1 μM) effectively inhibits EGF-stimulated (0.1 ng/mL) ERK phosphorylation. Blots represent representative of triplicate experiments.

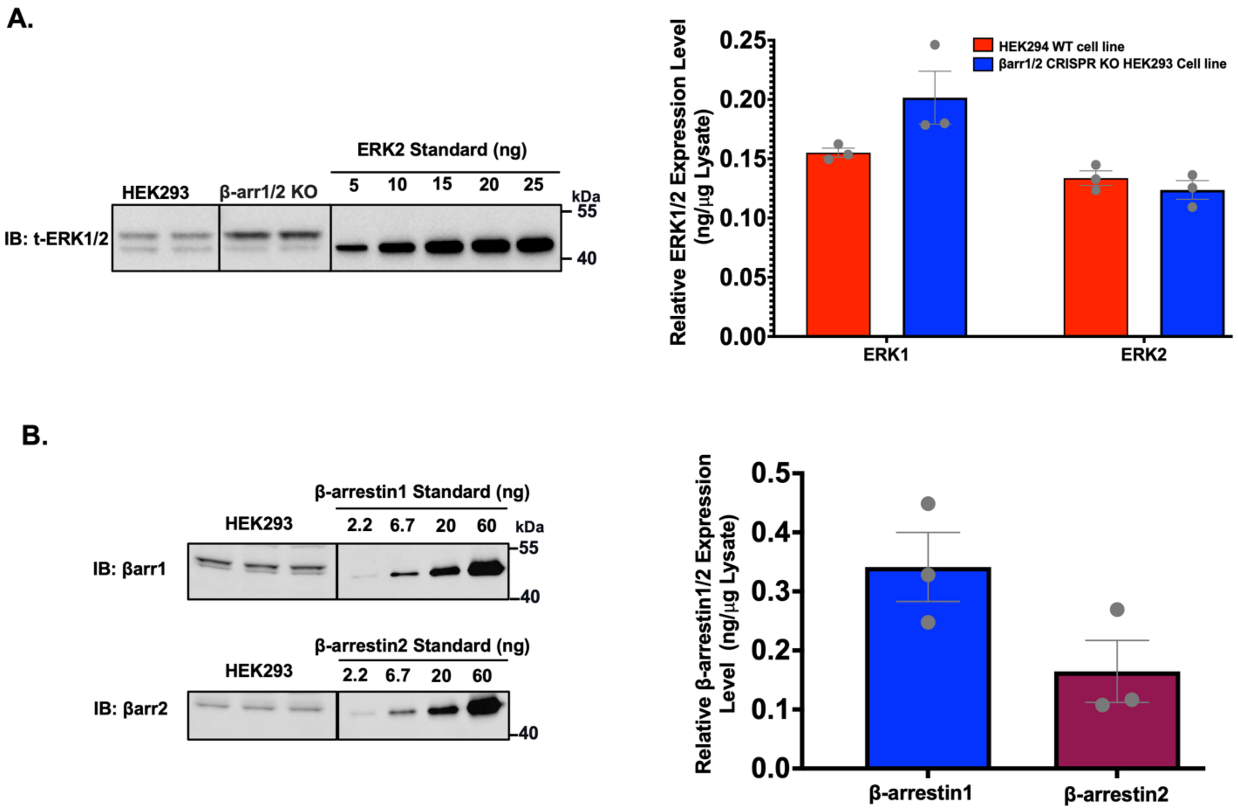


**Fig. S6. Analysis of Endogenous ERK1/2 and β-arrestin1/2 in Intact Cells. (*A*)** Analysis of endogenous ERK1/2 expression in HEK293 and CRISPR/Cas9 βarr1/2 KO HEK293 cell lines. **(*B*)** Analysis of endogenous βarr1 and 2 expression levels in HEK293 cell line. In ***A*** and ***B***, whole cell protein lysates were prepared from each cell line and protein concentrations were determined by BCA assay with BCA Protein Assay kit (Thermo Scientific™ Pierce™). Left panels show endogenous ERK1/2 **(*A*)** and βarr1/2 **(*B*)** expression levels as determined using a standard curve established from immunoblot analysis of recombinantly purified ERK2 (as in ***A***) and βarr1/2 (as in ***B***). Representative blots with approximate molecular weight of ERK1/2 **(*A*)** and βarr1/2 **(*B*)** are shown. Right panel bar graphs show a comparative analysis of the relative ERK1/2 **(*A*)** and βarr1/2 **(*B*)** expression levels quantified by densitometric analysis of immunoblots using established standards (*i.e.,* using respective recombinantly purified protein) in the indicated cell line. Data represent the mean ± SEM of triplicate experiments.

**SI References**


1. M. F. Favata *et al.*, Identification of a novel inhibitor of mitogen-activated protein kinase kinase. *J Biol Chem* **273**, 18623-18632 (1998).
2. T. Kinoshita *et al.*, Crystal structure of human mono-phosphorylated ERK1 at Tyr204. *Biochem Biophys Res Commun* **377**, 1123-1127 (2008).
3. B. Liu, Z. Guo, W. Gao, miR-181b-5p promotes proliferation and inhibits apoptosis of hypertrophic scar fibroblasts through regulating the MEK/ERK/p21 pathway. *Exp Ther Med* **17**, 1537-1544 (2019).
4. A. K. Shukla *et al.*, Visualization of arrestin recruitment by a G-protein-coupled receptor. *Nature* **512**, 218-222 (2014).
5. A. W. Kahsai *et al.*, Conformationally selective RNA aptamers allosterically modulate the beta2-adrenoceptor. *Nat Chem Biol* **12**, 709-716 (2016).
6. T. J. Cahill, 3rd *et al.*, Distinct conformations of GPCR-beta-arrestin complexes mediate desensitization, signaling, and endocytosis. *Proc Natl Acad Sci U S A* **114**, 2562-2567 (2017).
7. A. W. Kahsai *et al.*, Multiple ligand-specific conformations of the beta2-adrenergic receptor. *Nat Chem Biol* **7**, 692-700 (2011).
8. W. B. Asher *et al.*, GPCR-mediated beta-arrestin activation deconvoluted with single-molecule precision. *Cell* **185**, 1661-1675 e1616 (2022).
9. A. K. Shukla *et al.*, Structure of active beta-arrestin-1 bound to a G-protein-coupled receptor phosphopeptide. *Nature* **497**, 137-141 (2013).
10. A. H. Nguyen *et al.*, Structure of an endosomal signaling GPCR-G protein-beta-arrestin megacomplex. *Nat Struct Mol Biol* **26**, 1123-1131 (2019).
11. L. M. Luttrell *et al.*, Manifold roles of beta-arrestins in GPCR signaling elucidated with siRNA and CRISPR/Cas9. *Sci Signal* **11** (2018).
12. A. Tohgo, K. L. Pierce, E. W. Choy, R. J. Lefkowitz, L. M. Luttrell, beta-Arrestin scaffolding of the ERK cascade enhances cytosolic ERK activity but inhibits ERK-mediated transcription following angiotensin AT1a receptor stimulation. *J Biol Chem* **277**, 9429-9436 (2002).
13. L. M. Luttrell *et al.*, Activation and targeting of extracellular signal-regulated kinases by beta-arrestin scaffolds. *Proc Natl Acad Sci U S A* **98**, 2449-2454 (2001).
14. F. T. Lin, W. E. Miller, L. M. Luttrell, R. J. Lefkowitz, Feedback regulation of beta-arrestin1 function by extracellular signal-regulated kinases. *J Biol Chem* **274**, 15971-15974 (1999).
15. Y. Namkung *et al.*, Monitoring G protein-coupled receptor and beta-arrestin trafficking in live cells using enhanced bystander BRET. *Nat Commun* **7**, 12178 (2016).
